# Specific inhibition of CK2α from an anchor outside the active site†
†Electronic supplementary information (ESI) available: All experimental details, crystallographic data collection and refinement statistics, details of chemical synthesis, additional figures and tables. Data accessibility: all data supporting this study are included in the paper and provided as Supporting Information accompanying this paper. See DOI: 10.1039/c6sc02335e



**DOI:** 10.1039/c6sc02335e

**Published:** 2016-07-12

**Authors:** Paul Brear, Claudia De Fusco, Kathy Hadje Georgiou, Nicola J. Francis-Newton, Christopher J. Stubbs, Hannah F. Sore, Ashok R. Venkitaraman, Chris Abell, David R. Spring, Marko Hyvönen

**Affiliations:** a Department of Biochemistry , University of Cambridge , 80 Tennis Court Road , Cambridge CB2 1GA , UK . Email: mh256@cam.ac.uk; b Department of Chemistry , University of Cambridge , Lensfield Road , Cambridge , CB2 1EW , UK; c Medical Research Council Cancer Unit , University of Cambridge , Hutchison/MRC Research Centre , Hills Road , Cambridge CB2 0XZ , UK

## Abstract

The development of selective inhibitors of protein kinases is challenging because of the significant conservation of the ATP binding site. Here, we describe a new mechanism by which the protein kinase CK2α can be selectively inhibited using features outside the ATP site. We have identified a new binding site for small molecules on CK2α adjacent to the ATP site and behind the αD loop, termed the αD pocket. An elaborated fragment anchored in this site has been linked with a low affinity fragment binding in the ATP site, creating a novel and selective inhibitor (CAM4066) that binds CK2α with a *K*_d_ of 320 nM and shows significantly improved selectivity compared to other CK2α inhibitors. CAM4066 shows target engagement in several cell lines and similar potency to clinical trial candidate CX4945. Our data demonstrate that targeting a poorly conserved, cryptic pocket allows inhibition of CK2α *via* a novel mechanism, enabling the development of a new generation of selective CK2α inhibitors.

## Abstract 2

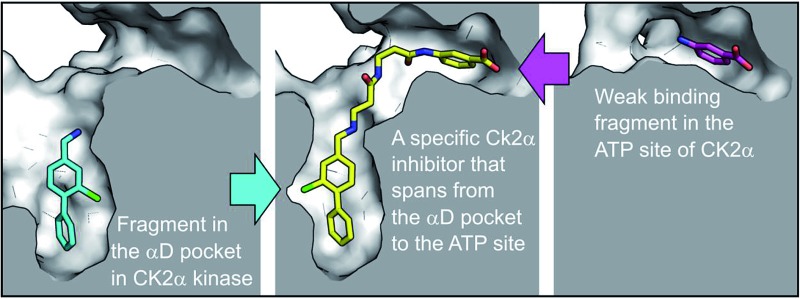
CAM4066, a specific CK2α kinase inhibitor, is anchored in the cryptic αD pocket outside the active site and inserts a “warhead” into the active site, blocking ATP binding and thereby inhibiting the kinase.

## Introduction

Protein kinases are key regulators of many cellular processes and are thus one of the main protein classes targeted in modern drug discovery.^1^ Kinase inhibitors typically bind in the deep ATP site, rendering the enzymes inactive by competing with the natural co-factor ATP. Despite many clinical success stories, the strong conservation of the ATP binding site across the kinase family makes it challenging to achieve selectivity.^2^ For example, imatinib inhibits not only its original target, Abl kinase, but eight other kinases with low nanomolar inhibitory constants.^3^ Therefore, inhibitors must derive selectivity from less conserved features of the kinase.^4^

CK2α is a highly-conserved, constitutively-active Ser/Thr kinase which is involved in the regulation of many cellular processes, including cell cycle progression, transcription and viral infections.^5–9^ CK2α provides pro-survival and anti-apoptotic effects on the cells, and is often overexpressed in cancer cells, promoting their proliferation by multiple mechanisms such as potentiation of the Akt pathway and activation of NF-Kβ.^10,11^ Cancer cells therefore rely on CK2α's activity to survive, becoming addicted to high levels of its non-oncogenic activity. This is particularly true for cells that are challenged with chemotherapeutic agents and the most promising uses for CK2α stem from these observations. Indeed, there have been many successful studies where CK2α inhibitors have been used in combination with established drugs and synergy has been demonstrated between them.^12–17^ In several recent examples, it has been shown that CK2α inhibitors can re-sensitize cells to treatments that they have become resistant to.^15–18^ For these reasons, selective inhibition of CK2α appears to be a promising strategy for cancer therapy.^19^ A number of potent ATP-competitive CK2α inhibitors^20,21^ have been shown to inhibit the growth of cancer cell lines and one of these, CX4945, has progressed to phase II clinical trials (Fig. S1†). CX4945 appears to be well-tolerated despite the target's ubiquitous role in cellular pathways. Although described as highly selective, CX4945 inhibits at least twelve other kinases with nanomolar IC_50_ values (Table S1†) and is more effective against Clk2 than CK2α.^22,23^ This results in clear cellular effects that are not linked to the inhibition of CK2α. Inhibition of Clk1, Clk2 and Clk3 by CX4945 has been shown to cause widespread, CK2α-independent, alteration of the alternative splicing of a significant number of genes.^23^ All CK2α inhibitors have this selectivity problem. Indeed, many of the inhibitors described as being selective against CK2α are also the most potent known inhibitors of DYRK3, HIPK3, DYRK2, HIPK4, DYRK4, DAPK3, Clk1, Clk2 and Clk3.^21,23^ Given the promise of CK2α as a therapeutic target and considering the limited selectivity achieved with current active site inhibitors, we wanted to investigate the possibility of developing more specific inhibitors by targeting sites outside of the conserved ATP site.

We report here the identification of a new binding site for small molecules on CK2α, adjacent to the ATP binding site, and the use of this site to develop a novel type of inhibitor of CK2α with high nanomolar affinity and with significantly improved selectivity compared to other known CK2α inhibitors. This proof of concept molecule has validated the use of this newly discovered site for the future development of higher affinity novel and selective inhibitors of CK2α.

## Results and discussion

**Fig. 1:**
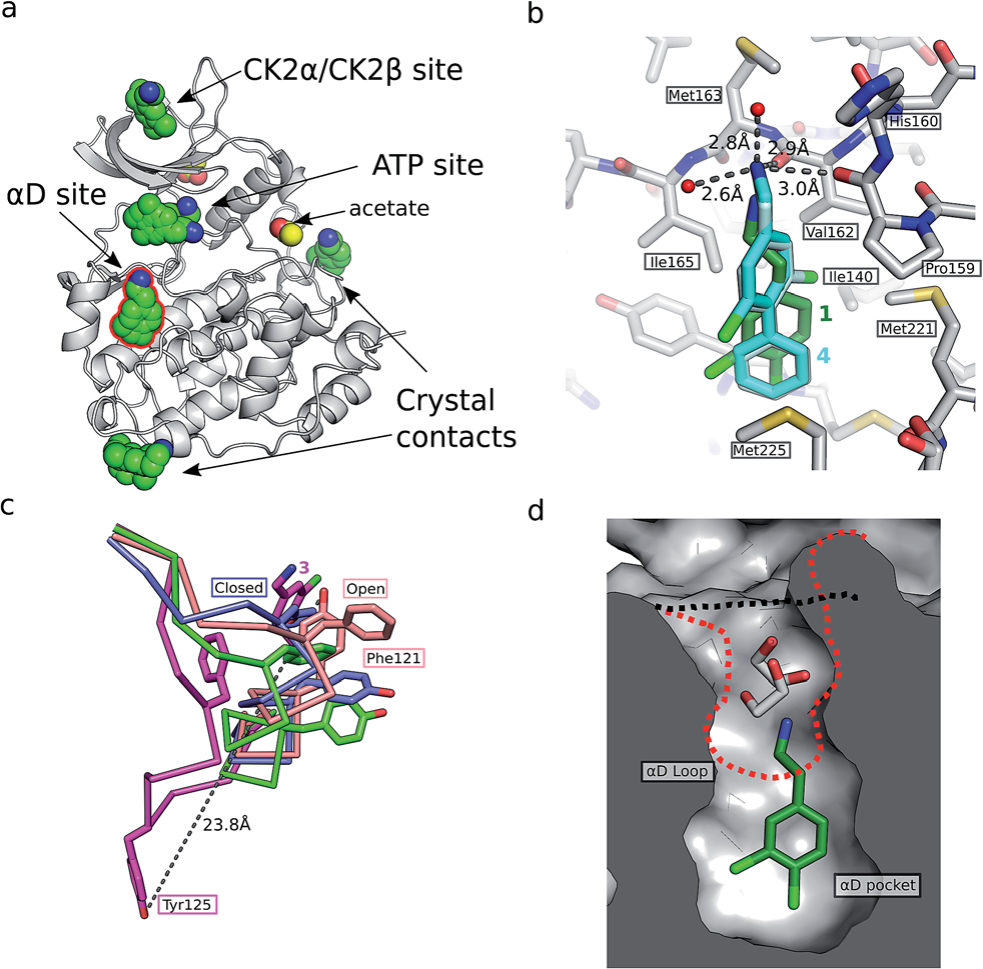
(a) The position of the seven molecules of 3,4-dichlorophenethylamine (**1**, green carbon and chlorine atoms) bound to CK2α (PDB : ; 5CLP), with the fragment bound in the αD pocket highlighted by a red outline. (b) A cross section of the αD pocket with **1** bound. The size of the αD pocket in the partially open CK2α structure, with ethylene glycol bound in the mouth of the pocket (PDB : ; 3WAR),^19^ is represented by the red dashed line, whereas the closed pocket (PDB : ; 3FWQ)^24^ is represented by the black dashed line. (c) A comparison of the binding mode of **1** (dark green carbons and lighter green chlorines) and **4** (cyan carbons and green chlorines, shown in both binding poses observed in the structure) with hydrogen bonds between the amine of **4** and backbone carbonyls and water molecules indicated with dashed lines, and side chains lining the αD site as sticks. (d) The conformations of the αD loop, Phe121 and Tyr 125 as induced by the binding of fragments shown for complexes with **1** (green, PDB : ; 5CLP) and **3** (magenta, PDB : ; 5CS6), for partially open apo form (pink, PDB : ; 3WAR)^25^ and for the closed apo enzyme (blue, PDB : ; 3FWQ).^24^ The largest rearrangement of the αD loop is highlighted with the dashed line indicating the shift of Tyr125 between the closed form and protein bound to **3** (shown as sticks).

In an attempt to develop chemical tools that target CK2α selectively, we used a high concentration crystallographic screen to identify novel fragments that could serve as starting points for chemical elaboration (see ESI† material for details). One of the fragments from this screen, 3,4-dichlorophenethylamine (**1**, Tables 1 and S2†), was observed to bind to CK2α at multiple different sites (Fig. 1a). Most interestingly, one of the bound fragments induced the opening of a pocket adjacent to the ATP site of the kinase (Fig. 1a). This new binding site, which we have termed the “αD pocket”, is located behind the αD helix (residues Asp120–Thr127) and is largely hydrophobic in character (Fig. 1b). This pocket is closed or only partially open in previously described crystal structures of CK2α, with either Phe121 or Tyr125 occupying the pocket (Fig. 1c). Two previously published structures of CK2α hint at the existence of this pocket. In one structure, of the partially open form of CK2α, two ethylene glycol molecules are bound at the entrance of the αD pocket (Fig. 1d).^25^ While in a recently published structure (PDB : ; 4UBA),^26^ a more open αD pocket can be observed, but no ligand is bound to it and no comment was made in the paper describing it. Nevertheless, the real size and potential of this pocket has only been revealed upon binding of our fragment (Fig. 1d). When **1** binds to the αD site it displaces Tyr125 from its normal position and releases the αD helix from the C-lobe, thus opening the αD pocket. The flexibility of the αD loop in CK2α has been noted before, and presumably this allows the pocket to open and the fragment to bind, forcing the helix from its typical position and creating the new binding site.^27^ Indeed, we observed that the loop is significantly more flexible than previously described.^28^ A large range of different loop conformations can exist such as the loop movement of 24 Å upon the binding of **3** (PDB : ; 5CS6, Fig. 1d). Moreover, during further crystallization studies, we identified a new crystal form of CK2α in which the αD pocket is filled by Leu124 (PDB : ; 5CVG, Fig. S3a and b†), and Phe121 occupies a shallow pocket in the hinge region leading to a large conformational change in that part of the ATP binding site (Fig. S3c†). This new conformation represents an inactive form of CK2α as the shifted residues in the hinge region would prevent the binding of ATP or GTP required for activity (Fig. S3d†) and may have implications in the regulation of CK2α.

**Table 1:** Summary of the compounds presented in this paper, their affinity towards CK2α and PDB accession codes for the corresponding crystal structures. (n.d. = not determined, n.b. = not binding)

	Structure	*K*_d_ (μM)	PDB
1	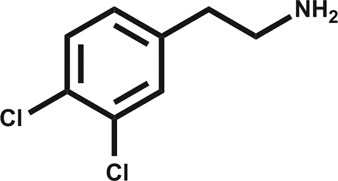	n.d.	5CLP
2	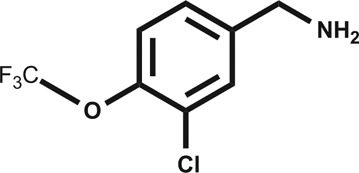	n.d.	5CVF
3	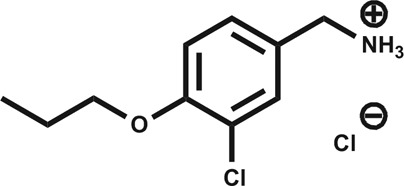	630	5CS6
4	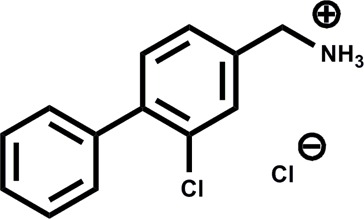	270	5CSH
5	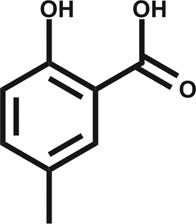	58	5CSP
6	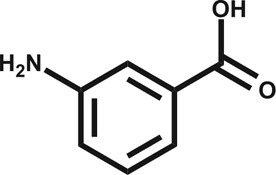	n.d.	5CSV
CAM4066 (**7**)	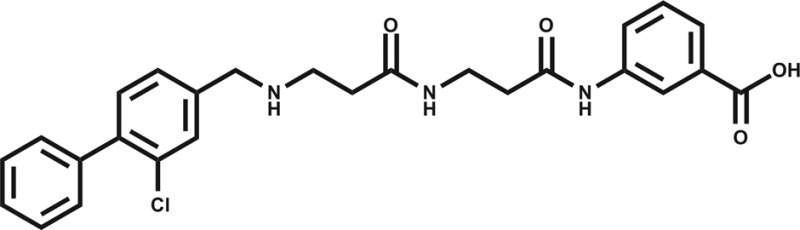	0.320	5CU3/; 5CU4
Pro-CAM4066 (**8**)	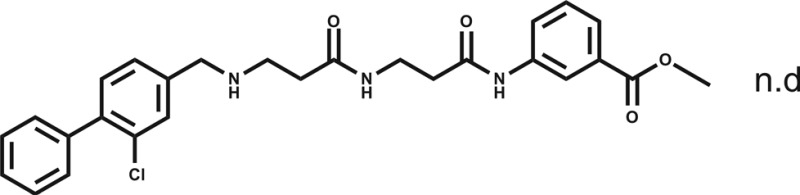	n.d	

Engagement of this pocket by small molecules offered the potential of a new approach for CK2α inhibition, which prompted us to further explore the druggability of this site. Optimization of the selectivity and affinity of **1** for the αD site *via* a series of analogues (**2** and **3**, Fig. S2†) led to **4** with a *K*_d_ of 270 μM (Fig. 1b, see ESI† methods for details of all the chemical synthesis). It was then hypothesized that it would be possible to link a fragment in the αD pocket with fragments that bound in the ATP site to produce a new kind of ATP competitive inhibitor. As the flexibility of the αD loop appears to be a unique feature of CK2α compared to other human kinases, it was predicted that linked inhibitors would have greater selectivity for CK2α over other kinases. This proof of concept molecule would validate this novel strategy for the inhibition of CK2α, which would enable the future development of novel and selective inhibitors of CK2α.

**Fig. 2:**
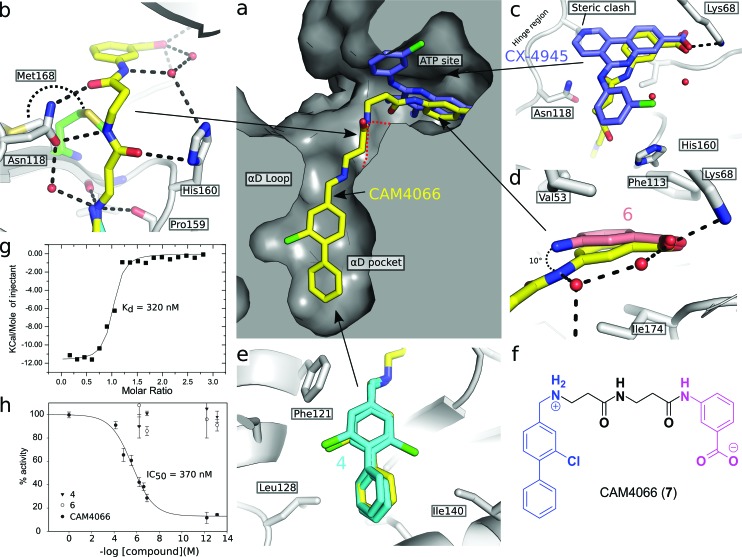
(a) Cross section view of the binding mode of CAM4066 (yellow) and CX4945 (dark blue) to the αD pocket and the ATP site of CK2α (PDB : 5CU4). (b) The hydrogen bonding network of CAM4066 with CK2α and three waters, is also shown. (c) A more detailed comparison of CAM4066 (yellow) and CX4945 (dark blue, PDB : ; 3NGA) binding in the ATP site. CAM4066 replicates the salt bridge of CX4945, however CX4945 also interacts with the hinge region which would make it unsuitable for linking to the αD pocket fragments due to the movement of Met163 and the hinge region. (d) A comparison between the binding of **6** and CAM4066 in the ATP site shows a tilt of 10° in order to link to the αD pocket. This indicates that the linker is not optimal. (e) A comparison between the binding of **4** (cyan) and CAM4066 (yellow) in the αD pocket shows that CAM4066 binds slightly higher in the pocket and only displays one binding mode, rather than the two modes displayed by **4**. (f) The structure of the linked compound CAM4066. The αD fragment is highlighted in blue and the ATP site fragment is highlighted in purple. (g) The isotherm of CAM4066 binding to CK2α (h) the inhibition of kinase activity by CAM4066, **4** and **6**. IC_50_ is shown for CAM4066 and error bars show the standard error from at least three replicates.

A separate X-ray screen of lower molecular weight fragments (see ESI† methods) then led to the discovery of twenty-five unique fragments binding in the ATP site. The highest affinity fragment from this screen was **5** (*K*_d_ 58 μM), however, **6** was chosen as the fragment to link to **4** as it showed no inhibition of phosphorylation at 500 μM or detectable binding by ITC, but was clearly observed to bind in the crystal structure. The idea being that the binding to CK2α should not be dominated by the interaction with the conserved ATP site but instead by the interaction with the αD site. Several fragments were found that interacted with the hinge region, however, these were later found to be unsuitable for progression as the movement of Met168 (Fig. 2b) upon binding of the linker occluded the binding of these fragments. The linking of **4** and **6** was investigated by the extension of **4** into the αD site with the synthesis of a series of compounds extending towards the ATP site (details to be published elsewhere) and eventually linking with **6**. The structure of the resulting compound CAM4066 (**7**, Fig. 2f), was determined in complex with CK2α (PDB : ; 5CU3 and ; 5CU4, Fig. 2a and S5†). These structures show that successful linking of binders in the αD site and the ATP site has created a new type of Ck2α inhibitor. CAM4066 maintains the key interactions seen in the fragment structures, with the “warhead” in the ATP site forming the expected salt bridge and hydrogen bond interactions. Likewise, the hydrophobic anchor in the αD pocket corresponding to **4** maintains its key interactions with the protein. The linker occupies a shallow groove connecting the two sites and is held in place by an elaborate network of hydrogen bonds (Fig. 2b). The ATP site warhead (**6**) is positioned in the same place as CX4945 would bind (Fig. 2c), but is tilted by 10° from the original fragment position (Fig. 2d). Like CX4945, CAM4066 makes a contact with Lys68, a conserved residue in many kinases, but it avoids making contacts with the hinge region.

The *K*_d_ of CAM4066 was determined by ITC as 320 nM (Fig. 2g), corresponding to almost a 1000-fold improvement on the highest affinity of the two starting fragments. CAM4066 also showed clear inhibition of CK2α kinase activity with an IC_50_ of 370 nM (Fig. 2h). This is a considerable improvement over the starting fragments (**4** and **6**), neither of which showed any inhibition at 500 μM.

**Fig. 3:**
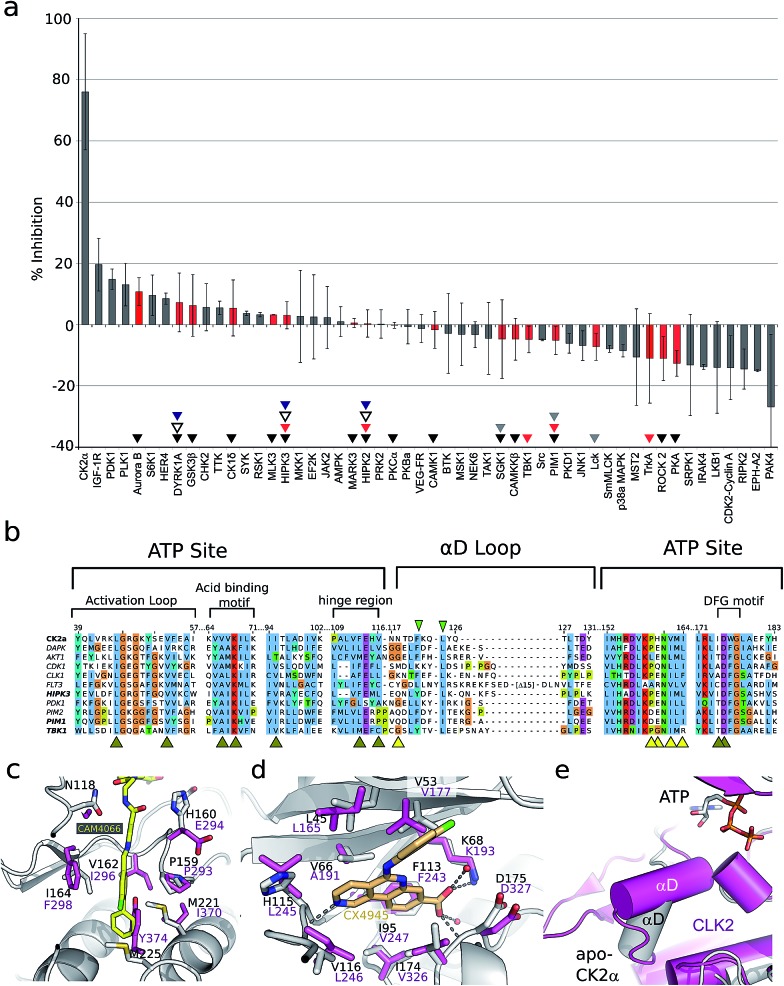
(a) The results from a kinase selectivity screen where CAM4066 was screened against 52 kinases at 2 μM. Data for kinases that are inhibited by other Ck2α inhibitors (CX4945: red triangles, D11: gray triangles, TF: black triangles, CX5279 blue triangles and **7h** white triangles) are highlighted with red bars. The error bars indicate the standard deviation from two replicates of the assay. (b) An alignment of the ATP site and the αD loop of CK2α and the kinases CX4945 inhibits with nM potencies. Alignment is coloured by conservation and the numbering on the top refers to human CK2α. Yellow and brown arrowheads under the alignment indicate residues in the CAM4066 and ATP binding sites, respectively. The green arrowheads highlight the two conserved residues in αD helix. (c) Superpositioning of Clk2 (purple, PDB : ; 3NR9) and CAM4066-bound CK2α (white) with residues in the αD pocket shown as sticks and labelled for both proteins. In the case of Clk2, the αD helix has been removed to allow for the view of the pocket. (d) View of the active sites of Clk2 (purple) and CX4945-bound CK2α (white, PDB : ; 3NGA). (e) Orientation of αD helix in unliganded CK2α and in Clk2 (purple).

As the aim of the project was to seek greater selectivity over existing inhibitors, compound CAM4066 was tested against a panel of 52 diverse kinases, chosen to provide a representative sampling of the human kinome and to include a number of kinases commonly inhibited by CK2α inhibitors. The kinases were assayed in the presence of 2 μM of CAM4066, ∼6 times above its IC_50_. At this concentration CK2α was inhibited by 75% (s.d. 19%), whereas no other kinases in the panel showed significant inhibition by CAM4066 (Fig. 3a). The greatest inhibition observed was 20% (s.d. 9%) against the kinase IGF-1R which is not considered as a significant off-target affect when the errors associated with this assay are taken into account. Analysis of the structure of IGF-1R also suggests that any off target inhibition is unlikely to be due to binding in the αD site (Fig. S7,† PDB : ; 5HZN). The Gini coefficient is routinely used to quantitatively compare the selectivity of kinase inhibitors that have been tested against different size screens.^29^ The Gini coefficient for CAM4066 was calculated to be 0.82 making it the most selective CK2α inhibitor reported to date (Table S2†). This provides a quantitative measure of the selectivity generated by targeting the αD pocket even though other compounds have been screened against larger panels of enzymes. For example, the Gini coefficient of the clinical trials candidate CX4945 is 0.615 and the Gini coefficient of CX5279, the most selective CK2α inhibitor previously reported, is 0.755 both of which are lower than that of CAM4066. A more extensive kinase screen could provide more definite evidence of the selectivity of CAM4066, but it would not demonstrate the uniqueness of αD pocket as it would provide no data on the binding mode causing the off target affects. As a proof-of-concept molecule, CAM4066 validates the novel concept of utilizing the poorly conserved αD pocket and demonstrates the benefit of using this pocket as an anchor point, thus gaining significant selectivity.

Only two residues, Phe121 and Leu123, in the αD loop of CK2α are conserved in any other kinase (Fig. 3b and S6†), and comparison of the structure of CK2α with these kinases shows that the pocket which binds CAM4066 (and the αD helix) is similarly less conserved (Fig. 3c–e and S7†). These results are in contrast to the reported selectivity profiles of the most “selective” ATP competitive CK2α inhibitors; CX4945,^22,30^ CX5279,^30^ D11,^31^ TF^32^ and compound **7h** by Dowling *et al.* ^21^ (Fig. S1a†). CX4945 has been shown to inhibit 26 other kinases by 70% or greater at 0.5 μM. At least twelve of these kinases were inhibited with low nanomolar IC_50_ values (screen size: 238 kinases at 0.5 μM).^22,30^ Indeed, the IC_50_ of CX4945 against Clk2 was reported to be 3.8 nM which is more potent than that reported against CK2α itself, in that study (Table S1†).^23^

Although Clk2 itself is not included within our panel, several closely related kinases in the CMGC family are (DYRK1A, HIPK2 HIPK3 and SRPK1). None of these related kinases are inhibited by CAM4066. Added to this information, analysis of the crystal structure of Clk2 indicates that it is unlikely to have an αD pocket that could accommodate CAM4066, especially with the side chain of Tyr374 blocking the bottom of that site (Fig. 3c) in contrast to the very related ATP site (Fig. 3d). Location of the αD helix of Clk2 is also significantly different from that of CK2α (Fig. 3e). Compound **7h** has impressive picomolar affinity for CK2α however when screened against a panel of 402 kinases at 0.1 μM, twelve were inhibited with IC_50_ values < 100 nM.^21^ CX5279, an analogue of CX4945, inhibits 4 other kinases by 70% or greater at 0.5 μM (102 kinases).^30^ D11 inhibits 61 other kinases by 70% or greater at 10 μM (354 kinases)^31^ and TF inhibits 7 other kinases by 70% or greater at 10 μM (61 kinases).^32^ HIPK2, HIPK3 and PIM1 are noteworthy as they are significantly inhibited by four (HIPK2-3) or 3 (PIM1) of these inhibitors,^21,30–32^ but show only 0% ± 4, and 3% ± 4 and –5% ± 5 inhibition, respectively, by CAM4066 at 2 μM. The off-target activities of these CK2α inhibitors are more significant when the drop-off in activity against cancer cell lines is considered. For all these inhibitors there is at least a 1000 fold reduction in activity against cell lines,^21,30–32^ compared to the inhibition/affinity against the isolated protein. The inhibitors are therefore being administered at concentrations higher than those that are known to cause significant inhibition of a large number of off target kinases. Thus, our initial data validates the novel strategy of targeting the newly discovered αD site. Furthermore, this data reveals the promise offered by further development of compounds targeting the αD site rather than the traditional method of targeting the ATP site.

**Fig. 4:**
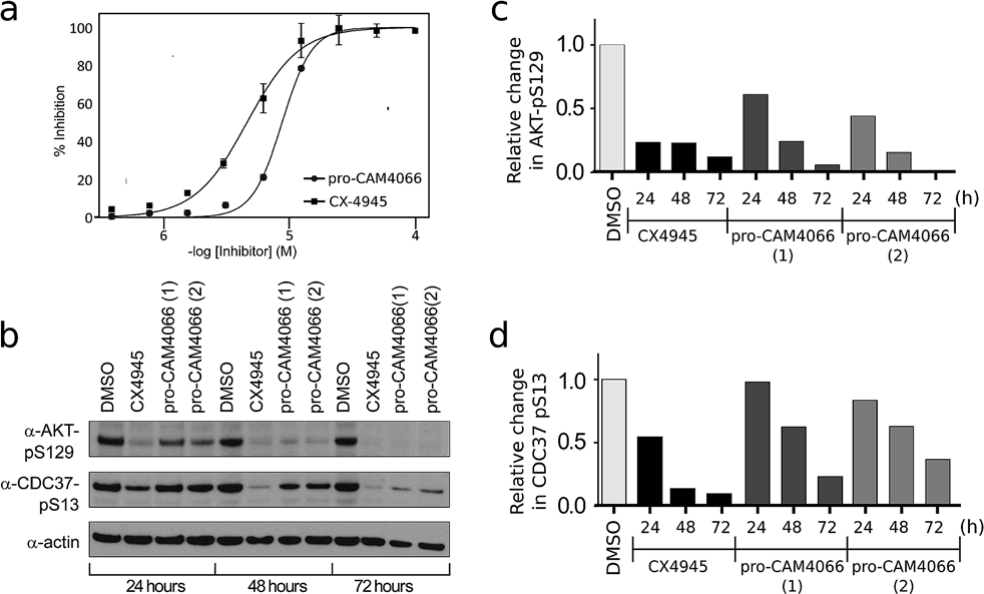
(a) Dose response curve for the inhibition of the growth of HCT116 cells by pro-CAM4066 and CX4945. CX4945 has a GI_50_ of 4.8 μM and pro-CAM4066 has a GI_50_ of 8.8 μM (graph for CX4945 shows the mean ± SEM of four independent experiments with each compound concentration in triplicate, and the graph for pro-CAM4066 shows the mean ± SEM of three experiments with each concentration in triplicate). (b) Western blot analysis showing the specific CK2 phosphorylation targets of AKT1 phosphoserine 129 and Cdc37 phosphoserine 13. HCT116 cells were treated with 2 × GI_50_ of CX4945 (10 μM) or **8** (20 μM) for 24, 48 or 72 hours. For pro-CAM4066 data is shown for two different experiments performed with different batches of inhibitor. (c) and (d) Show results of quantification of the Western blot shown in (b) for AKT1 phosphoserine 129 and Cdc37 phosphoserine 13, respectively. Data are normalised to actin loading control and to DMSO only sample.

The ability of CAM4066 to inhibit the growth of 3 different cancer cell lines was explored (Fig. 4) see ESI† methods. Initial attempts with CAM4066 showed no effect on cellular viability (data not shown). This was likely due to poor cellular penetration caused by the carboxylic acid and therefore the ester form of CAM4066, pro-CAM4066 (**8**), was used for the cellular assays, as esters are the most common form of pro-drug.^33,34^ This approach proved to be successful, and pro-CAM4066 showed good dose responses in HCT116, Jurkat and A549 cells, with a GI_50_ of 9, 6 and 20 μM, respectively (Fig. 4a and S8†).

This compares favorably with the clinical trial compound CX4945, which shows GI_50_ of 5, 5 and 17 μM against HCT116, Jurkat, and A549 cells. Although CX4945 has a considerably lower IC_50_ than CAM4066, the drop-off observed in the GI_50_ for pro-CAM4066 was only 19-60-fold, compared to the IC_50_; for CX4945 a 4000–15 000-fold drop-off in activity was observed. The target engagement was analyzed by following CK2α dependent phosphorylation of Ser129 of Akt1 and Ser13 of Cdc37. CX4945 showed very clear inhibition of the phosphorylation of both sites at all of the time points investigated at twice the GI_50_ concentration. In contrast to this, pro-CAM4066 showed only moderate (approximately 50%) inhibition of the phosphorylation of AKT1 Ser129 after 24 hours. However, at later time points of 48 and 72 hours, pro-CAM4066 inhibited the phosphorylation of this targets at a similar level to CX4945 (Fig. 4b–d). In contrast to this there was no inhibition of the phosphorylation of CDC37 after 24 hours, however again the inhibition did increase after 48 and 72 hours. This is in line with the reduced inhibition by CX4945 of the phosphorylation of CDC37 observed at 24 hours and may reflect a more stable phosphorylation site on CDC37 compared to AKT1 as observed previously.^35^ It is possible that this delay in inhibition, when compared to CX4945, is because the hydrolysis of pro-CAM4066 to release the active compound CAM4066 delays the achievement of pharmacologically active concentrations within cells which delays the onset of inhibition.

## Conclusion

In conclusion, we have used high concentration fragment soaks to probe the surface of CK2α leading to the identification of a cryptic pocket close to the ATP site. Fragments that bound in this cryptic pocket were linked successfully to a low affinity fragment in the ATP site. This quickly and efficiently generated a potent and highly selective inhibitor of CK2α, and validated the use of this cryptic pocket for the further development of CK2α inhibitors. Most significantly, we have fulfilled our initial aim and shown that it is possible to achieve higher levels of selectivity towards CK2α than previously reported for any inhibitor by interacting with features outside the active site. This can be rationalized by the low level of conservation around the αD pocket in kinases, compared to significantly higher similarity seen in the ATP site. The strategy of using such cryptic pockets outside of the active site to gain selectivity for inhibitors could be applied to other protein classes where selectivity is hard to achieve because of the high level of sequence conservation in the active site. This strategy could also be used to target orthologous proteins from pathogenic organisms to gain selectivity over host proteins.
