# Supplementary material for: Specific inhibition of CK2α from an anchor outside the active site
Source: Chem Sci. 2016 Jul 12;7(11):6839–45. doi: 10.1039/c6sc02335e (PMC5355960; doi:10.1039/c6sc02335e)

Electronic Supplementary Material (ESI) for Chemical Science.
This journal is © The Royal Society of Chemistry 2016

Supplementary information

Brear *et al.*, Inhibiting CK2α from an anchor outside the active site

Contents

Materials and methods 2
Protein expression and purification 2
X-ray crystallography 2
ITC 3
Kinase assays 3
Cell culture 3
Growth Inhibition assays 4
Western Blotting 4
Table S1. 5
Table S2. 6
Table S3. 7
Table S4. 10
Figure S1: 12
Figure S2. 14
Figure S3. 15
Figure S4. 16
Figure S5. 17
Figure S6. 18
Figure S7. 19
Chemical synthesis 20
General experimental details: 21
Synthesis of 3 22
Synthesis of 4 23
Synthesis of 7 24
NMR spectra of compounds 7 and 8_HCl 29

Materials and methods

Protein expression and purification

Four constructs of CK2α were used in this study. For ITC and kinase activity assays CK2α_WT was used (residues 2-329). For crystallization purposes three different constructs were used: CK2α_KA, CK2α_FP10 and CK2α_FP9. CK2α_KA (residues 2-329) contained four mutations designed to aid crystallization by reducing the overall charge of the protein; R21S, K74A, K75A and K76A. CK2α_FP10 contained one mutation (R21S) and an N-terminal extension GSMDIEFDDDADDDGSGSGSGSGS aimed at mimicking a substrate peptide for CK2α. CK2α_FP9 contained the same R21S mutation and the N-terminal extension GSMDIEFDDDADDDGSGSGSGSG. CK2α_FP10 and CK2α_FP9 were cloned into pHAT4 vector and CK2α_KA was cloned into pHAT2 vector to give constructs with cleavable His_6-tags.

Recombinant plasmids containing one of the four constructs (CK2α_WT/ CK2α_KA/ CK2α_FP10/ CK2α_FP9) were introduced into *Escherichia coli* BL21 (DE3) for protein production. Single colonies of the cells were grown in 6x1 L of 2xTY with 100 µg/mL ampicillin at 37 °C. Isopropyl thio-β-D-galactopyranoside (IPTG) was added to a final concentration of 0.4 mM to induce expression when the optical density at 600 nm reached 0.6. The cells were incubated overnight at 25 °C then harvested by centrifugation at 4,000 *g* for 20 minutes. The same extraction and purification procedure was used for all four constructs, with the exception that CK2α_KA used 350 mM NaCl in the buffer, whereas, CK2α_WT and CK2α_FP10/9 required 500 mM NaCl. The cell pellets were suspended in 20 mM Tris, 350/500 mM NaCl, pH 8.0) and lysed using a high pressure homogenizer. Protease inhibitor cocktail tablets (one tablet per 50 mL extract; Roche Diagnostics) and DNase I were then added. The crude cell extract was then centrifuged at 10,000 *g* for 45 minutes, the supernatant was filtered with a 0.22 µm filter. The soluble supernatant was applied on a Ni Sepharose Fast Flow6 column at pH 8.0, washed and eluted in 20 mM Tris pH 8.0, 350/500 mM NaCl, 200 mM imidazole. After overnight dialysis into 20 mM Tris, pH 8.0, 350/500 mM NaCl the N-terminal His_6-tag was cleaved overnight by TEV protease and passed through a second metal affinity column to remove uncleaved protein and the protease. The cleaved protein was further purified on a Sepharose Q HP anion-exchange column and the main peak fraction from this column was further purified by gel filtration on a Superdex 75 16/60 HiPrep column equilibrated with Tris 20 mM, pH 8.0, 350/500 mM NaCl. Pure protein was concentrated to 15 mg/mL and flash frozen in liquid nitrogen.

X-ray crystallography

CK2α_KA at 5 mg/mL in 20 mM Tris, pH 8.0, 350 mM NaCl, 1 mM DTT, and 25 mM ATP was crystallised with 112.5 mM MES pH 6.5, 35% glycerol ethoxylate and 180 mM ammonium acetate in a 1:1 ratio with a total volume of 2 µL by the hanging drop vapour-diffusion method. The fragments were soaked as singletons at 2-100 mM into these crystals for 15–20 h in 107 mM MES pH 6.5, 35% glycerol ethoxylate and 1.04 M ammonium acetate after which the crystals were cryo-cooled in liquid nitrogen for data collection.

CK2α_FP10 at 10 mg/mL in 20 mM Tris, pH 8.0, 500 mM NaCl, 4 mM DTT, 13 mM ATP, 2 mM phytic acid was crystallised with 107 mM MES, pH 6.5, 29% glycerol ethoxylate, 1.04 M ammonium acetate in a 1:1 ratio with a total volume of 2 µL by the hanging drop vapour-diffusion method. The fragments were soaked into the crystals of CK2α_FP10 for 15–20 h at 100 mM in 107 mM MES pH

6.5, 29% glycerol ethoxylate and 1.04 M ammonium acetate. The crystals were cryo-cooled in liquid nitrogen in the same solution for data collection. CK2α_FP9 at 5 mg/mL in 20 mM Tris, pH 8.0, 500 mM NaCl, 4 mM DTT, 8 mM ATP was crystallised with 92 mM MES, pH 6.5, 33% glycerol ethoxylate, 750 mM ammonium acetate in a 1:1 ratio with a total volume of 2 µL by the hanging drop vapour-diffusion method. The crystals were cryo-cooled in liquid nitrogen in the same solution for data collection.

Fragment screen to identify ATP site fragment was performed using 348-fragment Zenobia "ZEN-LIBRARY 1" library. The fragments were soaked as cocktails of four at 5 mM concentration for each fragments into CK2α_KA crystals in 15–20 h in 107 mM MES pH 6.5, 35% glycerol ethoxylate and 1.04 M ammonium acetate after which the crystals were cryo-cooled in liquid nitrogen for data collection.

X-ray diffraction data was collected at ESRF and Diamond synchrotron radiation sources, then processed using the pipedream package by Global Phasing Ltd; structures were solved by using programs from the CCP4 package.[25] Models were iteratively refined and rebuilt by using AutoBuster[26] and Coot programs. Ligand coordinates and restraints were generated from their SMILES strings using the Grade[27] software package. F_o-F_c electron density maps for all the ligands, calculated prior to ligand placement in the model, are shown in Figure S4 and stereo figures of CAM4066 density in Figure S5. All coordinates have been deposited to Protein Data Bank and accession numbers and data collection and refinement statistics are shown in Table S3 with crystallisation and soaking conditions listed in Table S4.

ITC

All ITC experiments were performed at 25 °C using a MicroCal itc200 instrument (GE Healthcare). CK2α_WT (20 mg/mL, 20mM Tris pH 8.0, 500 mM NaCl) was diluted in Tris buffer (200 mM Tris, 300 mM NaCl, 10% DMSO) and concentrated to 20-50 µM. Compounds in 100x stock solutions were diluted into the buffer ensuring that the DMSO concentrations were carefully matched. In a typical experiment CK2α_WT (40 µM) was loaded into the sample cell and 0.4-2.0 mM of the ligand was titrated in nineteen 2 µL injections of 2 s duration at 150 s intervals, with injector speed of 750 rpm. Heats of dilution were determined in identical experiments, but without protein in the cell. The data fitting was performed with a single site binding model using the Origin software package.

Kinase assays

The kinase assays were performed using the ADP-Glo™ kinase assay kit (Promega). 50 nM CK2α_WT was incubated in the kinase reaction buffer (40 mM Tris pH7.5, 200 mM NaCl, 20 mM $MgCl_2$, 0.1 mg/mL BSA, 25 µM ATP, 50 µM substrate peptide (RRRADDSDDDD, Enzo Life Sciences Inc.), 5% (v/v) DMSO) in the presence of different concentrations of the inhibitor at 25 °C for 40 min. 5 µL aliquots of the kinase reaction were quenched with 5 µL of ADP-glo™ solution. After another 40 min the kinase detection reagent was added and maintained at 25 °C for 30 minutes. The luminescence was recorded using a PHERAstar FS plate reader (BMG LABTECH) with an integration time of 1 s. Percentage inhibition was calculated relative to a DMSO control and a baseline measurement without ATP. All measurements were performed in triplicate. The IC_{50} curves were fitted using Sigma plot 11.0.

Cell culture

All cell lines used were obtained from ATCC and were supplied as mycoplasma free. HCT116 colon carcinoma cells were maintained in McCoy's 5A (1x) + Glutamax-I growth medium (Gibco, 36600-021) supplemented with fetal bovine serum (FBS, Gibco Life Technologies, 10270-106) at a final concentration of 10 %. A549 lung adenocarcinoma cells were cultured in Dulbecco Modified Eagle medium (DMEM) (1x) +Glutamax-I (Gibco Life Technologies, 31966-021) with 10% FBS. Jurkat T-cell lymphoblastic cells were cultured in RPMI 1640 (Gibco Life Technologies, 72400-021) growth medium supplemented with penicillin-streptomycin (Sigma, P0781) and FBS with the final concentrations of each being 1% and 10% v/v respectively. All cells were grown at 37 °C/ 5 % CO_2 in a humidified environment and all the assays were performed using these culturing conditions.

Growth Inhibition assays

Adherent cell lines (HCT116 and A549 cells) were seeded into flat-bottomed tissue culture 96-well plates in a volume of 150 µL of growth medium. HCT116 cells were seeded at 750 cells per well and A549 cells were seeded at 1000 cells per well. After 24 hours, compounds dissolved in DMSO were diluted in growth medium and were added to cells such that the final DMSO concentration was 1% (v/v) and the final volume in the well was 200 µL. Cells were then incubated in the presence compound for 72 hours before fixation.

Medium was removed from cells and 100 µL of cold 1% (v/v) trichloroacetic acid was added and the plates were incubated for 30 minutes at 4 °C after which the acid was removed and the plates were washed three times in tap water and left to dry at room temperature. The fixed cells were stained in a 0.057% sulforhodamine B/1% acetic acid solution (w/v) and incubated at room temperature with agitation for 30 minutes after which the dye was removed and the plates washed in 1% (v/v) acetic acid and left to dry. The dye was then solubilised in 10 mM Tris solution (pH8) and incubated for 30 minutes under agitation. The plates were then read on a PHERAstar plus plate reader (BMG Labtech) using the fluorescence intensity module (540-590 nm). Growth inhibition was calculated relative to DMSO controls and GI_{50} values were calculated using Graphpad Prism.

Jurkat cells were seeded (20,000 cells per well) in growth medium in a 96-well flat-bottomed plate and immediately dosed with compounds (dissolved in DMSO) such that the final volume in the well was 200 µL and 1% DMSO (v/v). Cells were incubated for a further 72 hours. After this time, 5% (v/v) of CellTitre-Blue reagent (Promega) was added to each well and incubated for a further 2 hours under normal tissue culture condition, described above. The fluorescence was then measured using the PHERAstar plus plate reader (BMG Labtech) using the fluorescence intensity module (540-590 nm). Growth inhibition was calculated relative to DMSO control and GI_{50} values were calculated using Graphpad Prism.

Western Blotting

HCT116 cells (2 ml) were seeded into 6-well tissue culture plates at a seeding density of $3x10^5$ cells/ml and cultured for 24 hours prior to the addition of compound. Compound was diluted in culture medium to the desired concentration and a final DMSO concentration of 1% (v/v). Cells were harvested by trypsination (growth medium was collected and included in the analysis), washed in PBS and the pellet collected. Cells were lysed using a NP-40 lysis buffer (50 mM Tris pH 8, 150 mM NaCl, 1% NP-40) with the addition of Proteoblock protease inhibitor (Fermentas), and the phosphatase inhibitors #2 and #3 (Sigma Aldrich) at the recommended concentrations. The cell pellet was incubated in lysis buffer on ice for 2 hours and then centrifuged for 10 minutes at 4 °C at

13000 rpm on a bench top centrifuge for 10 minutes and the supernatant collected and stored at -80 °C. Protein levels were quantified using the Pierce BCA protein assay kit (Pierce, Thermo Fisher Scientific).

A total of 30 µg of protein was loaded onto a 4-12% Bis-Tris gel (Invitrogen) and run for 1 hour at a constant 200 V. The gel was transferred onto PVDF membrane at 4 °C overnight at a constant 50 V. Transfer efficiency was confirmed by staining the membrane with Ponceau S (Sigma-Aldrich) after which it was incubated in blocking buffer (either 5 % Milk-TBS-0.1 % Tween 20 or 5 % BSA-TBS-0.1 % Tween 20) for 1 hour. Membranes were then incubated with either anti-AKT1 (phosphoS129) (Abcam, ab133458) or anti-Cdc37 (phospho S13) (Abcam, ab108360) antibodies diluted in 5% BSA-TBST for 24 hours at 4 °C. Anti-β actin was used as a loading control (Sigma-Aldrich, A5441) diluted in Milk-TBS-0.1% Tween 20. After washing, membranes were then incubated in HRP-labelled anti-rabbit antibody for 1 hour at room temperature and then visualised using ECL (GE Healthcare). Where membranes were stripped for re-probing, membranes were immersed in Restore Western Blot stripping buffer (Thermo Scientific) for 15 minutes at room temperature.

Table S1.

Off target effects of CX4945. List of kinases that CX-4945 is reported to inhibit with nanomolar IC_{50} values. Of particular note is CLK2, which is inhibited more potently by CX4945 than CK2α was under the same conditions.

Enzyme	IC_{50}	Reference
**CK2α**	14.7 nM	PLOS ONE (2014) 9:4
**CLK1**	82 nM	PLOS ONE (2014) 9:4
**CLK2**	3.8 nM	PLOS ONE (2014) 9:4
**CLK3**	90 nM	PLOS ONE (2014) 9:4
**CK2α**	1 nM	J.Med. Chem. (2011) 54:2:635
**DAPK3**	17 nM	J.Med. Chem. (2011) 54:2:635
**TBK1**	35 nM	J.Med. Chem. (2011) 54:2:635
**FLT3**	55 nM	J.Med. Chem. (2011) 54:2:635
**PIM1**	46 nM	J.Med. Chem. (2011) 54:2:635
**PIM1**	48 nM	Biorg. Med. Chem. Lett. (2011) 21;22;6687
**CLK3**	41 nM	J.Med. Chem. (2011) 54:2:635
**CDK1/Cyclin B**	56 nM	J.Med. Chem. (2011) 54:2:635
**HIPK3**	45 nM	J.Med. Chem. (2011) 54:2:635
**PIM2**	186 nM	Biorg. Med. Chem. Lett. (2011) 21;22;6687
**CDK2/Cyclin E**	1800 nM	Eur. J. Med. Chem. (2014) 78:217-224

Table S2.

Summary of the compounds presented and the relevant assay data associated with that compound. (n.d. = not determined, n.b. = not binding)

	Structure	K_D (μM)	IC_{50} (μM)	GI_{50} (μM) Jurkat	GI_{50} (μM) HCT116	GI_{50} (μM) A549	Gini Coeff.
1	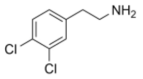	n.d.	n.d.	n.d.	n.d.	n.d.	n.d.
2	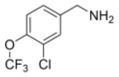	n.d.	n.d.	n.d.	n.d.	n.d.	n.d.
3	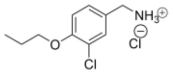	630	>500	n.d.	n.d.	n.d.	n.d.
4	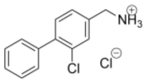	270	>500	n.d.	n.d.	n.d.	n.d.
5	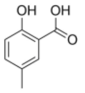	58	n.d.	n.d.	n.d.	n.d.	n.d.
6	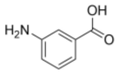	n.d.	>500	n.d.	n.d.	n.d.	n.d.
CAM4066 (7)	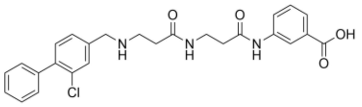	0.320	0.370	>500	>500	>500	0.82
pro-CAM4066 (8)	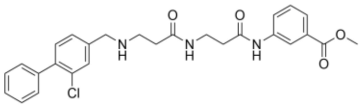	n.d.	n.d	5	7	20	n.d.
CX-4945	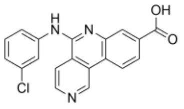	0.001	0.001	4	4	15	0.62
CX-5279	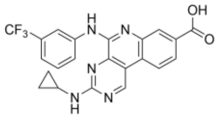	n.d	0.001	3.1	>10	n.d	0.755

Table S3.

Data collection and refinement statistics.

Description	CK2α + **1**	CK2α + **2**	CK2α + **3**	CK2α + **4**
PDB code	5CLP	5CVF	5CS6	5CSH
**Data collection**				
Wave length (Å)	0.9762	0.9393	0.9202	0.9188
Space group	C 2 2 2_1	P 2_1	C 2 2 2_1	C 2 2 2_1
Cell dimensions				
a, *b*, *c* (Å)	65.0, 69.0, 334.3	58.7, 45.9, 62.9	64.5, 68.8, 335.9	64.3, 69.2, 334.0
α, β, γ (°)	90.0, 90.0, 90.0	90.0 111.9 90.0	90.0, 90.0, 90.0	90.0, 90.0, 90.0
Resolution (Å)	167.17-1.68 (1.689-1.684)	55 - 1.63 (1.66 - 1.63)	167.94 -1.88 (1.886 - 1.880)	83.5 -1.59 (1.595 -1.589)
R_{sym} or R_{merge}	0.077 (0.877)	0.069 (0.752)	0.063 (1.002)	0.032 (0.563)
I / σI	14.3 (2.2)	10.4 (1.5)	16.0 (2.0)	20.3 (2.0)
Completeness (%)	100.0 (100.00)	97.8 (94.4)	100.0 (100.0)	99.5 (99.4)
Redundancy	6.6 (6.3)	3.3 (3.1)	8.1 (8.3)	3.7 (3.6)
Beamline	DLS: I03	ESRF: ID14-4	DLS: I02	DLS: I02
**Refinement**				
Resolution (Å)	167.17-1.68 (1.72-1.68)	55 - 1.63 (1.66 - 1.63)	167.94-1.88 (1.92-1.88)	83.5-1.58 (1.63-1.58)
No. reflections	564270 (5542)	123930 (5626)	496000 (5279)	367334 (3626)
R_{work} /	0.1778 (0.2183)	0.2009 (0.2639)	0.2056 (0.2414)	0.1930 (0.2171)
R_{free}	0.1933 (0.2336)	/0.2090 (0.2766)	0.2158 (0.2623)	0.2067 (0.2286)
No. atoms				
Protein	5594	2799	5518	5545
Ligand/ion	188	84	73	133
Water	448	211	242	401
B-factors (Å^2)				
Protein	30.3	27.4	71.4	44.4
Ligand/ion	45.7	47	72.3	54.9
Water	40.2	34	62.7	40.5
R.m.s. deviations				
Bond lengths (Å)	0.014	0.015	0.014	0.014
Bond angles (°)	1.57	1.84	1.65	1.70

*Values in parentheses are for highest-resolution shell.

Table S3 continued. Data collection and refinement statistics.

Description	CK2α + **5**	CK2α + **6**	CK2α + **7**	CK2α + **7**
PDB code	5CSP	5CSV	5CU3	5CU4
**Data collection**				
Wave length (Å)	0.92	0.9173	0.9173	0.9795
Space group	P 2_1	P 2_1	C 2 2 2_1	P 2_1
Cell dimensions				
a, *b*, *c* (Å)	58.7, 45.5, 63.7	58.5, 45.4 63.4	65.3, 66.3, 333.6	58.6, 46.2 62.6
α, β, γ (°)	90.0, 111.9, 90.0	90.0, 111.7, 90.0,	90.0, 90.0, 90.0	90.0, 112.7, 90.0
Resolution (Å)	54.47 - 1.5 (1.501-1.496)	58.92 - 1.37 (1.380 - 1.375)	55.6 - 1.79 (1.93- 1.787)	46.19 - 1.56 (1.59 - 1.56)
R_{merge}	0.024 (0.283)	0.028 (0.449)	0.045 (0.638)	0.060 (0.647)
$I / \sigma I$	29.3 (5.2)	19.0 (2.0)	20.20 (2.1)	11.3 (1.5)
Completeness (%)	78.13 (16.9)	99.60 (98.4)	100,0 (99.6)	96.9 (76.9)
Redundancy	3.2 (2.2)	3.2 (2.6)	6.3 (4.9)	3.2 (2.4)
Beamline	DLS: I04-1	DLS: I04-1	DLS: I04-1	DLS: I02
**Refinement**				
Resolution (Å)	54.47-1.50 (1.53-1.50)	58.91-1.36 (1.40-1.36)	55.60-1.78 (1.83-1.78)	36.08-1.56 (1.60-1.56)
No. reflections	125113 (185)	207389 (1560)	437940 (3329)	138379 (4081)
R_{work}	0.1636 (0.2264)	0.1795 (0.2124)	0.1835 (0.2171)	0.1744 (0.2371)
R_{free}	0.1857 (0.2092)	0.1950 (0.2070)	0.2018 (0.2288)	0.1951 (0.2810)
No. atoms				
Protein	2810	2792	5495	2821
Ligand/ion	19	18	101	46
Water	293	269	402	303
B-factors (Å^2)				
Protein	21.2	24.2	39.3	22
Ligand/ion	23.2	28.2	39.9	27.4
Water	31.2	33.7	46.9	32.1
R.m.s. deviations				
Bond lengths (Å)	0.014	0.014	0.014	0.015
Bond angles (°)	1.6	1.68	1.61	1.67

*Values in parentheses are for highest-resolution shell.

Description	CK2α	apo-CK2α	apo-CK2α
PDB code	5CVG	5CVH	5CU6
**Data collection**			
Wave length (Å)	0.9795	0.9795	0.9795
Space group	P 2_1	C 222_1	P 2_1
Cell dimensions			
a, *b*, *c* (Å)	57.6, 44.1, 62.0	64.5, 68.7 333.1	58.5, 45.5, 63.6
α, β, γ (°)	90. 110.4 90.0	90.0 90.0 90.0	90.0 111.2 90.0
Resolution (Å)	35.13-1.25 (1.254-1.250)	166.55 - 1.85 (1.855 - 1.848)	54.55 - 1.36 (1.364-1.360)
R_{merge}	0.042 (0.446)		
$I / \sigma I$	14.8 (2.1)	20.6 (2.2)	0.042 (0.446)
Completeness (%)	90.8 (48.3)	100.0 (99.5)	99.7 (98.3)
Redundancy	3.2 (2.4)	6.6 (6.8)	3.3 (3.0)
Beamline	DLS: I04	DLS: I04	DLS: I04
**Refinement**			
Resolution (Å)	35.13-1.25 (1.28-1.25)	166.55-1.84 (1.89-1.84)	54.55-1.35 (1.39-1.35)
No. reflections	231929 (960)	420167 (4378)	220382 (1925)
R_{work}	0.1532 (0.2021)	0.2031 (0.2568)	0.1866 (0.2250)
R_{free}	0.1698 (0.2126)	0.2196 (0.2654)	0.2090 (0.2334)
No. atoms			
Protein	2837	5535	2805
Ligand/ion	16	132	39
Water	306	219	256
B-factors (Å^2)			
Protein	17.9	60.3	23.9
Ligand/ion	21.9	98.1	34.2
Water	28.5	52.9	30.5
R.m.s. deviations			
Bond lengths (Å)	0.014	0.015	0.014
Bond angles (°)	1.62	1.81	1.75

*Values in parentheses are for highest-resolution shell.

Table S4.

Crystallisation and ligand soaking conditions

PDB code	5CLP	5CVF	5CS6	5CSH	5CSP	5CSV	5CU3	5CU4	5CVG	5CVH	5CU6
Ligand	**1**	**2**	**3**	**4**	**5**	**6**	**7**	**10**	**apo**	**apo**	**apo**
Ligand code	**42J**	**54Z**	**K82**	**54E**	**54G**	**GAB**	**54S**	**54S**	**n/a**	**ATP**	**ATP**
Construct	FP10	KA	FP10	FP10	KA	KA	FP10	KA	FP9	FP10	KA
Crystallisation:	**A**	**B**	**A**	**A**	**B**	**B**	**A**	**B**	**A**	**B**	**A**
Cryo-cooling:	50 mM **1**, in **A**	50 mM **2**, in **A**	100 mM **3**, in **A**	100 mM **4**, in **A**	5 mM **5**, in **D**	25 mM **6**, in **D**	1 mM **10**, in **D**	2 mM **10**, in E	**E**<br>**A**	**E**<br>**A**	**E**<br>**A**
Soaking:	50 mM **1**, in **A**	50 mM **2**, in **A**	100 mM **3**, in **A**	100 mM **4**, in **A**	5 mM **5**, in **D**	25 mM **6**, in **D**	1 mM **10**, in **D**	2 mM **10**, in **E**	**n/a**	**n/a**	**n/a**

Condition A:	107mM Mes pH 6.5, 29% glycerol ethoxylate, 1 M ammonium acetate
Condition B:	112.5mM Mes pH 6.5, 35% glycerol ethoxylate, 180 mM ammonium acetate
Condition C:	92 mM Mes pH 6.5, 33% glycerol ethoxylate, 750 mM ammonium acetate
Condition D:	107mM Mes pH 6.5, 29% glycerol ethoxylate, 1 M ammonium acetate, 5% DMSO
Condition E:	2 mM 10, 107mM Mes pH 6.5, 29% glycerol ethoxylate, 1 M ammonium acetate, 30% DMSO

Table S5.

Breakdown of the ITC data. The enthalpy and entropy contributions towards the K_D values.

Compound	K_d (µM)	ΔH (cal/mol)	ΔS (cal/mol/deg)	Comment
**3**	630	-5047	-2.28	Forces the αD loop into a fully open position.
**4**	270	-2363	8.33	Forces the αD loop into a partially open conformation.
**5**	58	-7722	-6.51	ATP site only
**7**	0.320	-11600	-9.36	αD site and ATP site

Figure S1:

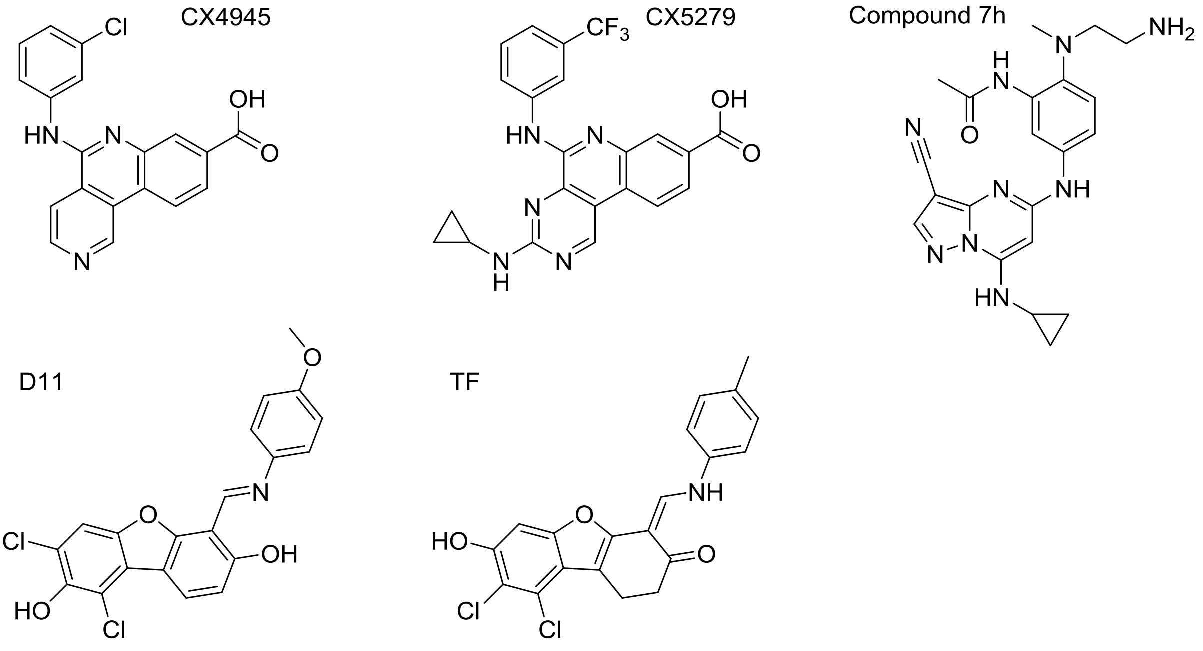

Known CK2α binders. A. The structure of three inhibitors of CK2α (CX4945, CX5279, Compound 7h D11, TF,) that have the most extensive selectivity data reported in the literature.

Figure S2.

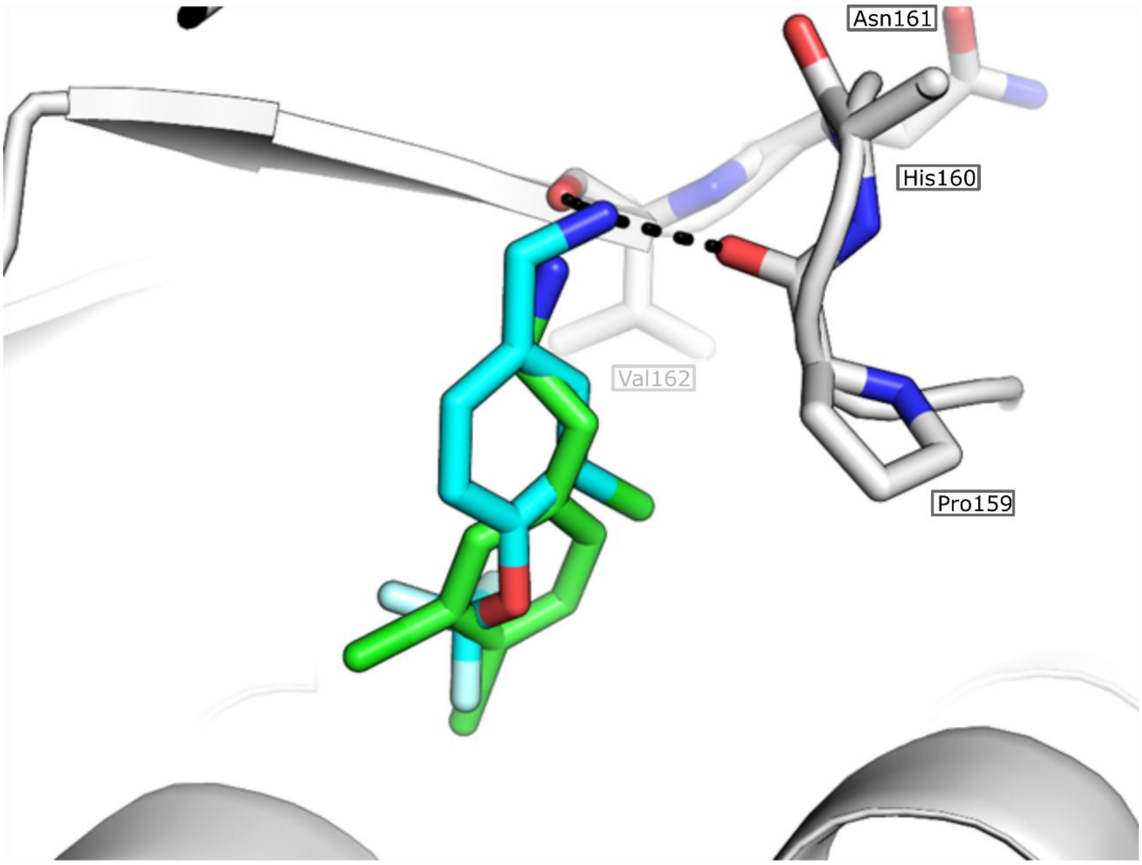

Comparison of the binding mode of 1 and 2. Both compounds interact with the backbone carbonyl of Pro159 and Val 162, however, **2** sits higher up in the pocket. The CF_3 group of **2** occupies a similar position to the dichloro-phenyl of **1**.

Figure S1

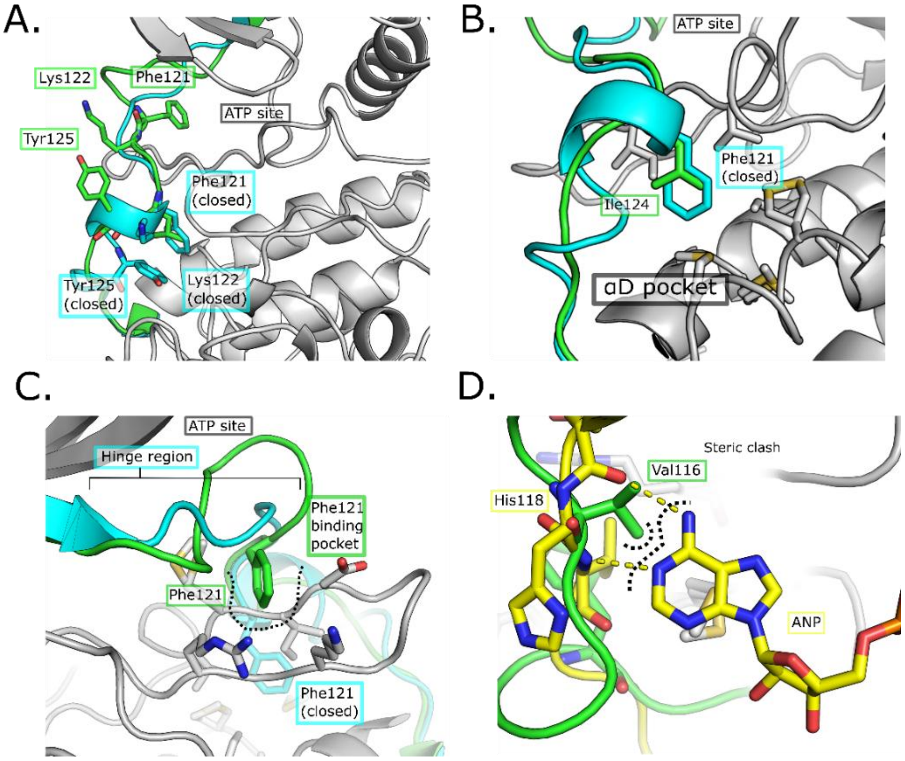

Crystal structure of CK2α with a novel conformation of the αD loop. **A**. The differences in the conformation of the αD loop between CK2αFP9 (green) and the published closed structure of CK2α (pdb:3FWQ)[22]. Lys122 and Tyr125 occupy the terminal of the substrate binding channel in the closed structure, whereas, in the novel loop conformation Lys122 and Tyr125 have moved out of the substrate binding channel. Similarly Phe121 in the closed structure fills the αD pocket but in the CK2α_FP9 structure it occupies a new pocket in the hinge region. **B**. In the novel conformation the αD pocket is filled with Ile124, whereas in the closed form it is filled with Phe121. **C**. In the CK2α_FP9 structure of the αD loop Phe121 occupied a shallow pocket previously in the hinge region. This led to a large distortion of this region which is important for the binding of ATP. **D**. An overlay of the hinge region of the open ADP bound structure (yellow) and the Novel conformation (green) showed that it was an inactive conformation due to the steric clashes between ATP and the hinge region.

Figure S4.

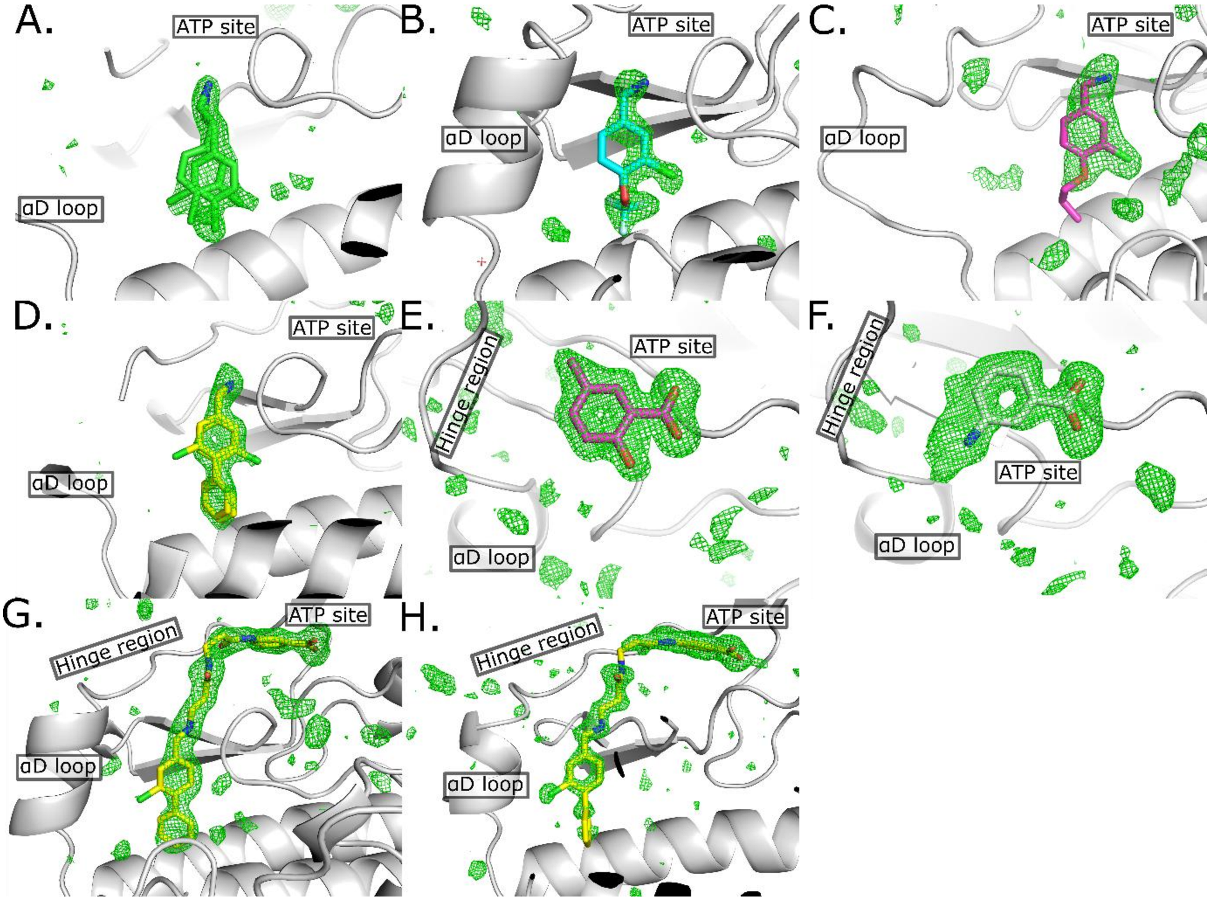

F_o-F_c density map contoured at 3σ for compounds 1-7 bound to CK2α. **A**. **1** bound to CK2α_FP10, **B**. **2** bound to CK2α_KA, **C**. **3** bound to CK2α_FP10, **D**. **4** bound to CK2α_FP10, **E**. **5** bound to CK2α_KA, **F**. **6** bound to CK2α_KA, **G**. **7** bound to CK2α_FP10, **M**. **7** bound to CK2α_KA.

Figure S5.

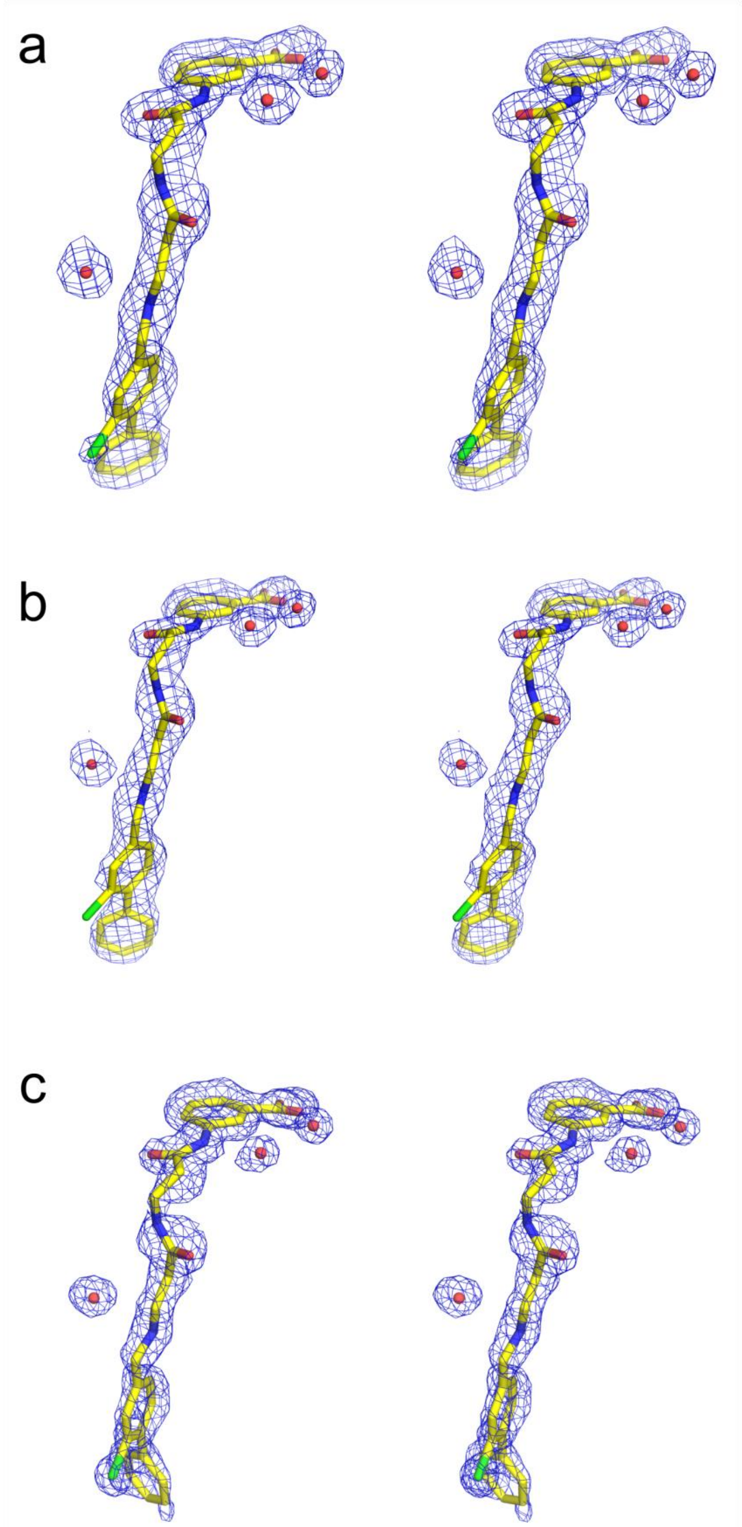

Stereo images of CAM4066 electron densities. (a) Weighted 2FoFc electron density map for CAM4066 (yellow carbons) and directly coordinated water molecules in CK2a_FP10 crystal form, countered at 1σ. (b) As (a) but for the second CK2α-CAM4066 complex in the asymmetric unit. (c) As (a) and (b), but for the CAM4066 complex in CK2α-KA crystal form.

Figure S6.

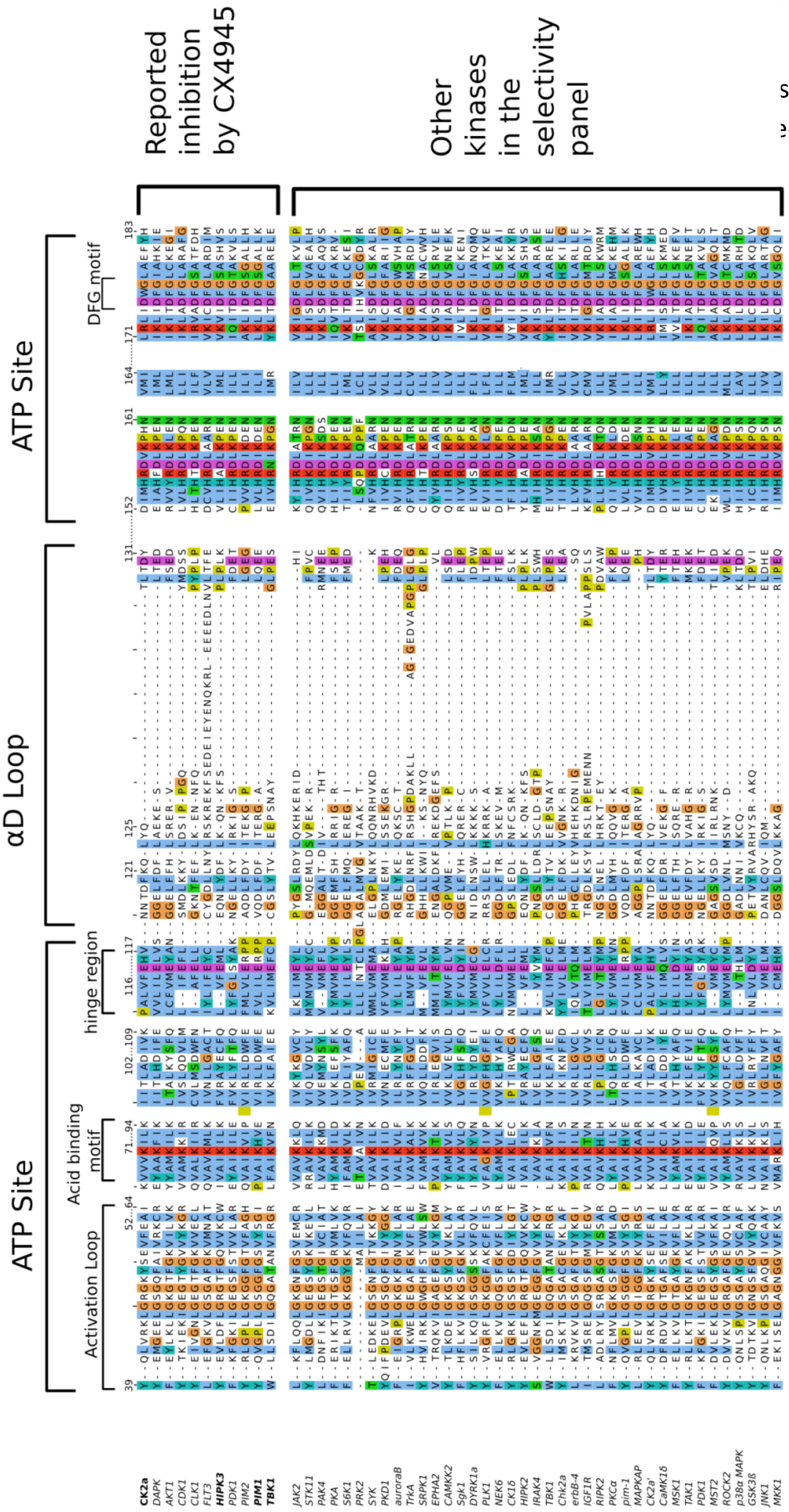

Sequence ali[illegible]s showing only key areas of the [illegible]ed in the selectivity panel.

Figure S7.

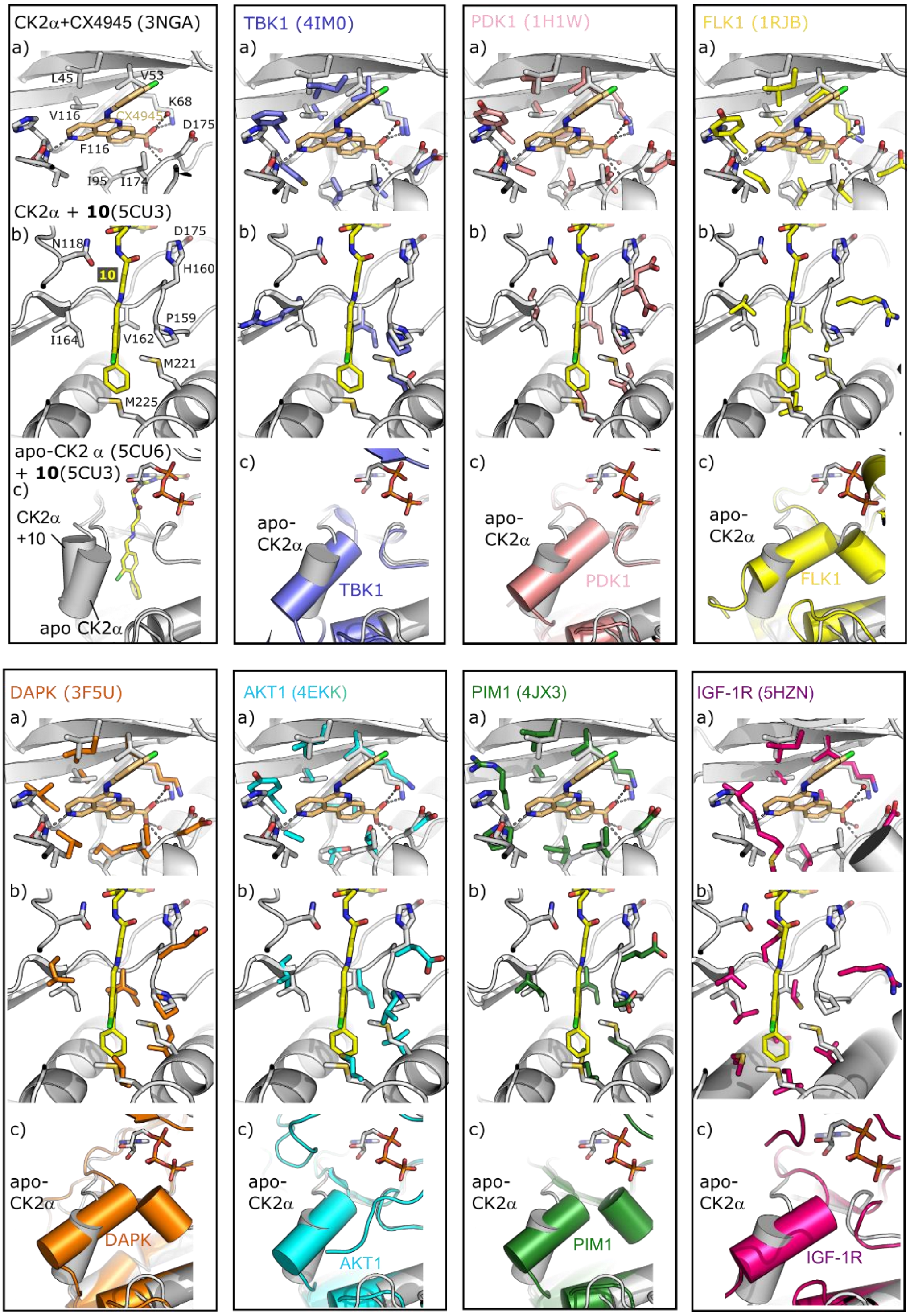

Structural comparison of CK2α and other kinases. Each panel shows a comparison of CK2α and another kinase as labeled at the top. In all figures CK2α structure is shown in light gray and the other kinase in color. (a) Comparison in the ATP site showing CX4945 bound to CK2α. (b) Comparison in the αD pocket and residues binding to CAM4066. (c) Comparison of position of the αD helices in the kinases.

Figure S8.

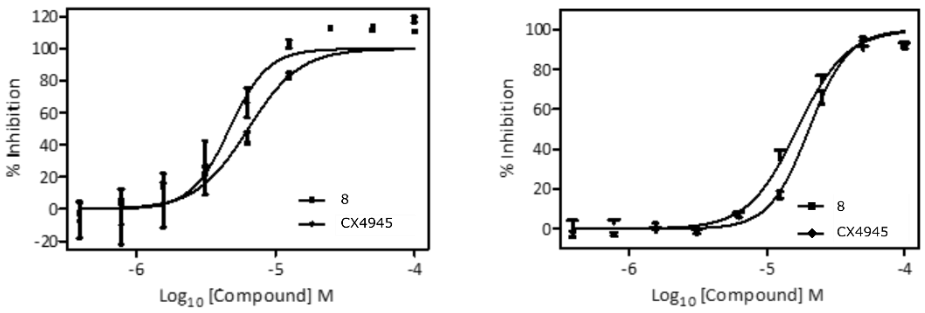

Growth inhibition data from Jurkat and A549 cells. (**a**) Growth inhibition curves of Jurkat cells in the presence of different concentration of compound **8** (squares) and CX4945 (diamonds). (**b)** Similar data for A549 cells. The data shown is from two experiments with each concentration point in triplicate. The GI_{50} values for compound **8** are 6.3 and 20.2 µM in Jurkat and A549, respectively and the GI_{50} values for CX4945 are 4.7 and 16.6 µM.

Chemical synthesis

Compounds **3**, **4**, **7** and **8** were synthesized as outlined in schemes S1 and S2.

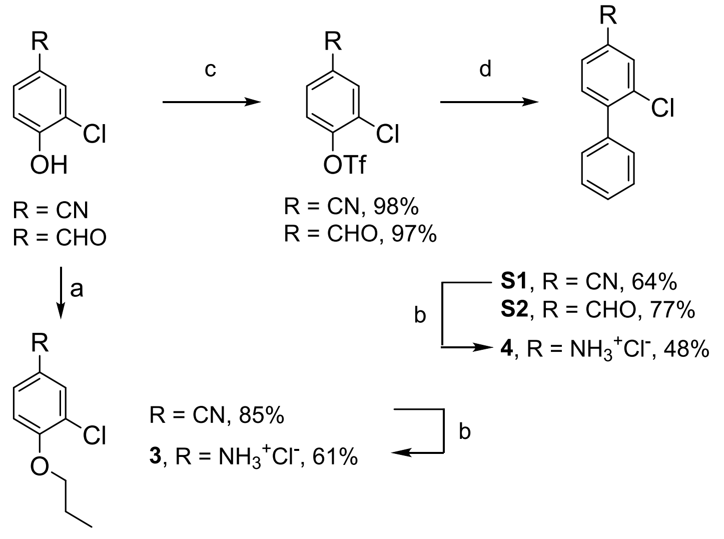

Scheme S1: a)1-bromopropane, K_2CO_3, DMF; b) (i) $LiAlH_4$, $AlCl_3$, Et_2O; (ii) 2N HCl in Et_2O; c) Tf_2O, Py, CH_2Cl_2, 0°C; d) phenylboronic acid, $Pd(PPh_3)_4$, Na_2CO_3, H_2O/DME, reflux.

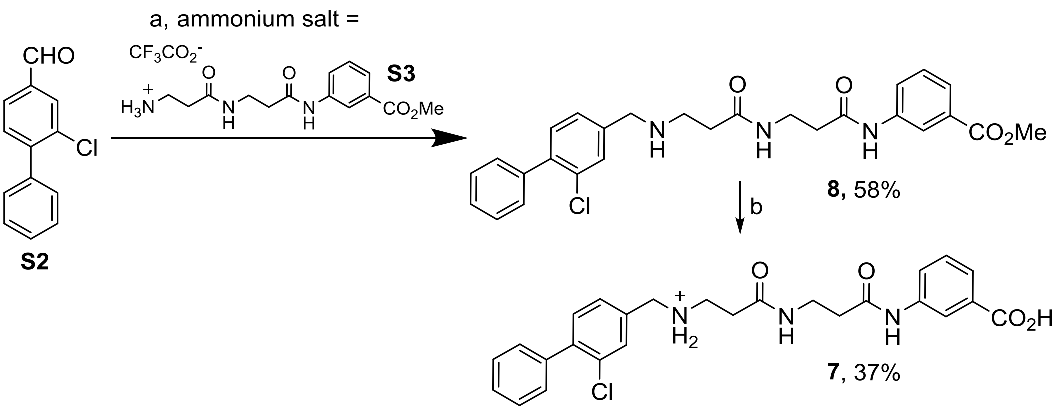

Scheme S2: a) ammonium salt, $Na(AcO)_3BH$, Et_3N, MeOH; b) (i) LiOH, H_2O/THF, (ii) 4N HCl in dioxane.

The ammonium salt **S3** was prepared as described in scheme S3.

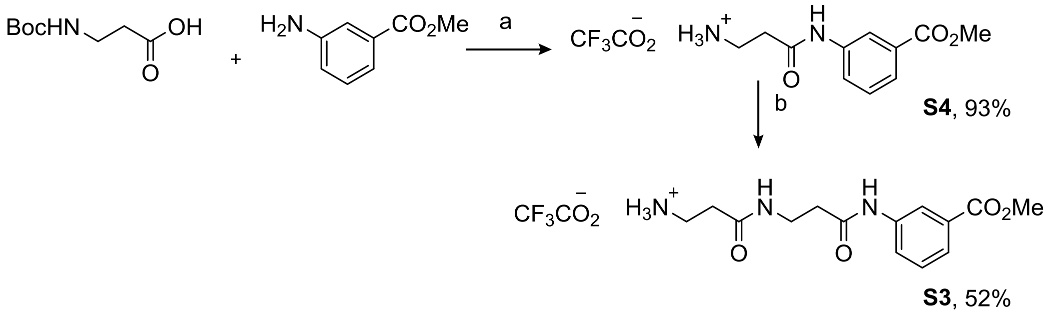

Scheme S3: a) (i) DCC, DMAP, CH_2Cl_2; (ii) TFA/CH_2Cl_2; b) (i) Boc-β-alanine, HATU, DMAP, DMF; (ii) TFA/CH_2Cl_2.

General experimental details:

Solvents: Except as otherwise indicated, reactions were carried out using oven-dried glassware under nitrogen with dry, freshly distilled solvents. THF was distilled from CaH_2 and $LiAlH_4$ in the presence of triphenylmethane. Diethyl ether was distilled from CaH_2 and $LiAlH_4$. CH_2Cl_2 and MeOH were distilled from CaH_2. All other solvents were used as obtained from commercial sources.

Materials: all reagents were used as obtained from commercial sources. 3-Chloro-4-propoxybenzonitrile,[1] 2-chloro-4-cyanophenyl trifluoromethanesulfonate,[2] and 2-chloro-4-formylphenyl trifluoromethanesulfonate[3] were synthesized according literature procedures.

TLC: All reactions were monitored by thin layer chromatography (TLC) using glass plates precoated with Merck silica gel 60 F254. Visualization was by the quenching of UV fluorescence (λ_{max} = 254 nm) or by staining with ninhydrin. Retention factors (R_f) are quoted to 0.01.

Chromatography: Flash column chromatography was carried out using slurry-packed Merck 9385 Kieselgel 60 silica gel under a positive pressure of nitrogen.

Semi-Preparative HPLC: HPLC purification was performed on an Agilent 1260 Infinity system fitted with a Supelcosil ABZ+Plus column (250 mm x 21.2 mm, 5 µm) using linear gradient systems (solvent A: 0.1% (v/v) TFA in water, solvent B: 0.05% (v/v) TFA in acetonitrile) at a flow rate of 20 mL·min^{-1}.

Infrared (IR): IR spectra were recorded on a Perkin-Elmer Spectrum One FT-IR spectrometer using a Diamant/KRS5 ATR. Selected absorption maxima (ν_{max}) are reported in wavenumbers (cm^{-1}).

---

[1] Chinnagolla, R. K.; Pimparkar, S.; Jeganmohan, M. *Chem. Commun.* **2013**, *49*, 3146.

[2] Dawson, M. I.; Xia, Z.; Liu, G.; Fontana, J. A.; Farhana, L.; Patel, B. B.; Arumugarajah, S.; Bhuiyan, M.; Zhang, X.-K.; Han, Y.-H.; Stallcup, W. B.; Fukkushi, J.-I.; Mustelin, T.; Tautz, L.; Su, Y.; Harris, D. L.; Waleh, N.; Hobbs, P. D.; Jong, L.; Chao, W.; Schiff, L. J.; Sani, B. P. *J. Med. Chem.* **2007**, *50*, 2622.

[3] Yang, C.; Edsall, R.; Harris, H. H.; Zhang, X.; Manas, E. S.; Mewshaw, R. E. *Bioorg. Med. Chem.* **2004**, *12*, 2553.

NMR: Nuclear magnetic resonance spectra were recorded using an internal deuterium lock on Bruker DPX 400 (400MHz), Bruker Avance 400 QNP Ultrashield (400 MHz), Bruker Avance 500 BB ATM (500 MHz) and Bruker Avance 500 Cryo Ultrashield (500 MHz) spectrometers. Chemical shifts (δ) are referenced to the solvent residual peak and are quoted in ppm to the nearest 0.01 ppm for δ_H and to the nearest 0.1 ppm for δ_C. Coupling constants (*J*) are reported in Hertz to the nearest 0.1 Hz.

HRMS: High resolution mass spectrometry was carried out with a Micromass Q-TOF or a Waters LCT Premier Mass Spectrometer using electrospray ionisation [ESI].

Melting points: These data were collected on a BÜCHI B-545 and are uncorrected.

Compound preparation and characterization:

Synthesis of 3

(3-Chloro-4-propoxyphenyl)methanaminium chloride (3)

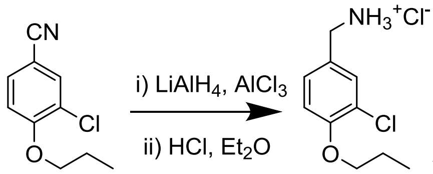

To a suspension of lithium aluminium hydride (117 mg, 3.07 mmol) in anhydrous diethyl ether (6 mL), under nitrogen, was added aluminium chloride (408 mg, 3.07 mmol). The reaction mixture was cooled to 0 °C and stirred for 10 min. The reaction was allowed to warm to rt and the 3-chloro-4-propoxybenzonitrile (300 mg, 1.53 mmol) was added portionwise as a solution in diethyl ether. The reaction was stirred at rt for 30 min and then heated to 40 °C for 18 h. The resultant suspension was allowed to cool to rt, diluted with ethyl acetate, saturated aqueous potassium sodium tartrate tetrahydrate was added and the mixture was stirred for 1h. The reaction mixture was poured into 2M aqueous sodium carbonate and extracted with ethyl acetate (3×). The combined organic extracts were dried ($MgSO_4$) and concentrated under reduced pressure to yield the desired product (285 mg, 93%). The crude amine (285 mg, 1.42 mmol) was converted to the hydrochloric salt adding hydrochloric acid (7.10 ml, 2 M in diethyl ether) to a CH_2Cl_2 solution of the amine (0.4M). The reaction mixture was stirred for 1h at rt and the precipitate was filtered off and washed with diethyl ether to yield the product as an off-white crystalline solid (222 mg, 66%).

Mp = 246-249 °C; **δ_H /ppm** (500 MHz, d_6-DMSO): 8.36 (3H, br s), 7.60 (1H, d, *J* = 2.2 Hz), 7.40 (1H, dd, *J* = 8.5, 2.2 Hz), 7.17 (1H, d, *J* = 8.5 Hz), 4.03 (2H, t, *J* = 6.4 Hz), 3.94 (2H, s), 1.79-1.70 (2H, m), 0.99

(3H, t, *J* = 7.4 Hz); **δ_C /ppm** (126 MHz, d_6-DMSO): 154.0 (C), 130.7 (CH), 129.3 (CH), 127.0 (C), 121.1 (C), 113.7 (CH), 70.1 (CH2), 41.1 (CH2), 21.9 (CH2), 10.3 (CH3); **ν_{max}/cm^{-1}:** 2963, 1610, 1506; **HRMS** (ESI+) *m/z* found $[M+H]^+$ 200.0832, $C_{10}H_{15}NO^{35}Cl^+$ required 200.0837.

Synthesis of 4

2-chloro-[1,1'-biphenyl]-4-carbonitrile (S1)

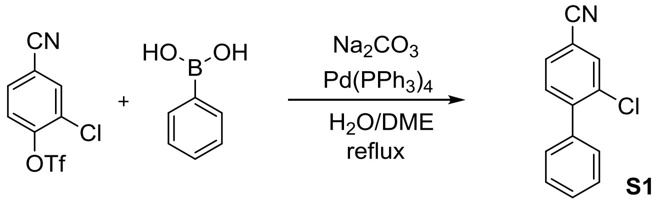

A mixture of 2-chloro-4-cyanophenyl trifluoromethanesulfonate (2.74 g, 9.59 mmol), phenylboronic acid (1.51 g, 12.4 mmol), sodium carbonate (28.7 mmol, 2M aqueous solution) and ethylene glycol dimethyl ether (60.0 mL) was degassed for 10 min. Tetrakis(triphenylphosphine)-palladium (554 mg, 0.479 mmol) was added and the mixture was heated to reflux for 3.5 h. The mixture was allowed to cool to rt and poured into water, then extracted with ethyl acetate (3x), washed with brine, dried ($MgSO_4$) and the solvent was evaporated under reduced pressure. The crude residue was purified by column chromatography eluting with *n*-hexane/ethyl acetate 95:5 to yield the product as a white solid (1.31 g, 64%).

R_f = 0.63 (*n*-hexane/ethyl acetate 80:20); **Mp** = 78-79 °C; **δ_H /ppm** (500 MHz, d_6-DMSO): 8.18 (1H, dd, *J* = 1.6, 0.2 Hz), 7.91 (1H, dd, *J* = 8.0, 1.6 Hz), 7.62 (1H, dd, *J* = 8.0, 0.3 Hz), 7.54-7.44 (5H, m); **δ_C /ppm** (126 MHz, d_6-DMSO): 144.6 (C), 137.3 (C), 133.4 (CH), 132.4 (CH), 132.3 (C), 131.3 (CH), 129.0 (CH), 128.6 (CH), 128.4 (CH), 117.5 (C), 111.9 (C); **ν_{max}/cm^{-1}:** 3065, 2230, 1595, 1501, 1474; **HRMS** (ESI+) m/z found $[M+H]^+$ 214.0412, $C_{13}H_9N^{35}Cl^+$ required 214.0418.

(2-Chloro-[1,1'-biphenyl]-4-yl)methanaminium chloride (4)

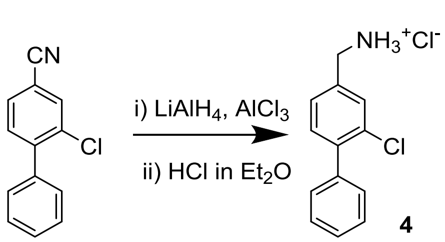

To a suspension of lithium aluminium hydride (106 mg, 2.81 mmol) in anhydrous diethyl ether (4.68 mL), under nitrogen, was added aluminium chloride (374 mg, 2.81 mmol). The reaction mixture

was cooled to 0 °C and stirred for 10 min. The reaction was allowed to warm to rt and the 2-chloro-[1,1'-biphenyl]-4-carbonitrile **S1** (300 mg, 1.40 mmol) was added portionwise as a solution in diethyl ether. The reaction was stirred at rt for 30 minutes and then heated to 40 °C for 18 h. The resultant suspension was allowed to cool to rt and ethyl acetate and saturated aqueous potassium sodium tartrate tetrahydrate were added and the mixture stirred for 1h. The reaction mixture was poured into 2 M aqueous sodium carbonate and extracted with ethyl acetate (3×). The combined organic extracts were dried ($MgSO_4$) and concentrated under reduced pressure to give the primary amine (283 mg, 93%). The crude amine (24.0 mg, 0.110 mmol) was converted to the hydrochloride salt adding hydrochloric acid (0.550 mL, 2 M in diethyl ether) to a CH_2Cl_2 solution of the amine (0.4 M). The reaction was stirred for 1 h and the precipitate was filtered off and washed with diethyl ether to yield the product as a white crystalline solid (19.9 mg, 71%).

Mp = 270-273 °C; **δ_H /ppm** (500 MHz, d_6-DMSO): 8.45 (3H, br s), 7.75 (1H, app d, *J* = 1.7 Hz), 7.54 (1H, dd, *J* = 7.9, 1.7 Hz), 7.51-7.46 (3H, m), 7.45-7.40 (3H, m), 4.09 (2H, s); **δ_C /ppm** (126 MHz, d_6-DMSO): 139.8 (C), 138.2 (C), 135.3 (C), 131.6 (CH), 131.2 (C), 130.3 (CH), 129.1 (CH), 128.3 (CH), 128.1 (CH), 128.0 (CH), 41.3 (CH2); **ν_{max} /cm^{-1}:** 3420, 2913, 1607, 1514; **HRMS** (ESI+) *m/z* found $[M+H]^+$ 218.0729, $C_{13}H_{13}N^{35}Cl^+$ required 218.0737.

Synthesis of 7

2-Chloro-[1,1'-biphenyl]-4-carbaldehyde (S2)

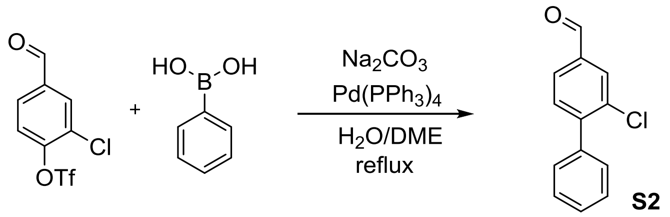

A mixture of the 2-chloro-4-formylphenyl trifluoromethanesulfonate (6.35 g, 22.0 mmol), phenylboronic acid (3.49 g, 28.6 mmol), sodium carbonate (66.0 mmol, 33.0 mL of a 2 M aqueous solution) and ethylene glycol dimethyl ether (138 mL) was degassed for 30 min. Tetrakis(triphenylphosphine)-palladium (1.27 g, 1.10 mmol) was added and the mixture was heated to reflux for 3.5 h. The mixture was allowed to cool to rt and poured into water, then extracted with ethyl acetate (3x). The combined organic extracts were dried ($MgSO_4$) and the solvent evaporated under reduced pressure. The crude residue was purified by column chromatography eluting with a 98:2 to 90:10 *n*-hexane/ethyl acetate gradient to yield the product as a white solid (3.69 g, 77%).

R$_f$ = 0.20 (*n*-hexane/ethyl acetate 95:5); **Mp** = 96-98 °C; **δ_H /ppm** (400 MHz, $CDCl_3$): 10.02 (1H, s), 7.99 (1H, d, *J* = 1.6 Hz), 7.83 (1H, dd, *J* = 7.8, 1.6 Hz), 7.53 (1H, d, *J* = 7.8 Hz), 7.50-7.40 (5H, m); **δ_C /ppm** (101 MHz, $CDCl_3$): 190.8 (CH), 146.5 (C), 138.3 (C), 136.6 (C), 133.8 (C), 132.2 (CH), 131.2 (CH), 129.3 (CH), 128.6 (CH), 128.4 (CH), 128.0 (CH); **ν_{max}/cm^{-1}:** 2835, 1697, 1597; **HRMS** (ESI+) *m/z* found $[M+H]^+$ 217.0414, $C_{13}H_{10}O^{35}Cl^+$ required 217.0420.

3-((3-(Methoxycarbonyl)phenyl)amino)-3-oxopropan-1-aminium trifluoroacetate (S4)

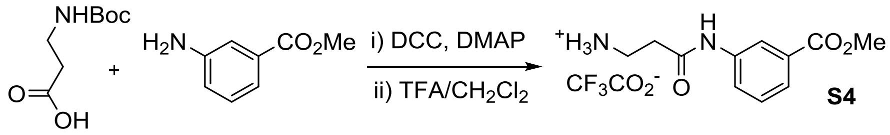

To an ice-cooled solution of Boc-β-alanine (1.51 g, 8.00 mmol) in CH_2Cl_2 (40.0 mL), DCC (1.65 g, 8.00 mmol) and DMAP (97.7 mg, 0.800 mmol) were added. After 10 min stirring methyl 3-aminobenzoate (1.45 g, 9.60 mmol) was added and the reaction mixture stirred overnight at rt, filtered and the solid was washed with CH_2Cl_2 (x2). The combined filtrate was washed with 2 M HCl and brine, dried ($MgSO_4$) and the solvent was removed under reduced pressure. The crude material was then purified by column chromatography eluting with a 99:1 to 98:2 CH_2Cl_2/MeOH gradient (R$_f$ = 0.56, CH_2Cl_2/MeOH 95:5) affording the *N*-Boc protected intermediate (2.45 g, 95%). This material (2.10 g, 6.51 mmol) was treated with a 1:1 mixture of CH_2Cl_2/TFA at 0 °C and stirred for 30 min at rt. Volatiles were evaporated and the product was obtained as a white solid (2.19 g, 99%).

Mp = 117-119 °C; **δ_H /ppm** (400 MHz, *d*$_6$-DMSO): 10.41 (1H, s), 8.33 (1H, t, *J* = 1.7 Hz) 7.90-7.76 (4H, m), 7.65 (1H, dt, *J* = 7.8, 1.1 Hz), 7.47 (1H, t, *J* = 8.0 Hz), 3.16-3.06 (2H, m), 2.73 (2H, t, *J* = 6.7 Hz); **δ_C /ppm** (101 MHz, *d*$_6$-DMSO): 168.7 (C), 166.0 (C), 139.2 (C), 130.1 (C), 129.3 (CH), 124.0 (CH), 123.6 (CH), 119.7 (CH), 52.2 (CH3), 34.9 (CH2), 33.2 (CH2); **ν_{max}/cm^{-1}:** 2989, 2935, 1722, 1672, 1566, 1463, 1205, 1129, 801, 755, 722; **HRMS** (ESI+) *m/z* found $[M+H]^+$ 223.1068, $C_{11}H_{15}N_2O_3^+$ required 223.1077.

3-((3-((3-(Methoxycarbonyl)phenyl)amino)-3-oxopropyl)amino)-3-oxopropan-1-aminium trifluoroacetate (S3)

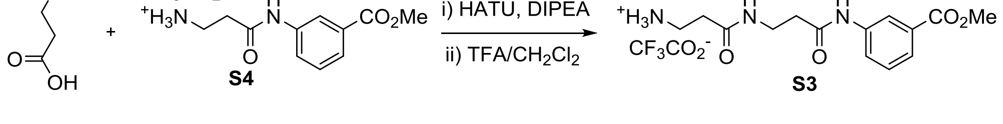

To an ice-cooled solution of β-alanine (189 mg, 1.00 mmol) in DMF (10.0 mL), were added HATU (380 mg, 1.00 mmol) and NMM (220 μL, 2.00 mmol). After 10 min stirring the ammonium salt **S4**

(269 mg, 0.800 mmol) was added and the reaction mixture was stirred overnight at rt. The solution was diluted with CH_2Cl_2 and washed with 10% aqueous citric acid and brine (x3). The organic layer was dried ($MgSO_4$), concentrated under reduced pressure and the crude product was purified by flash chromatography eluting with a 98:2 to 95:5 CH_2Cl_2/MeOH gradient (R_f = 0.20, CH_2Cl_2/MeOH 95:5) affording the *N*-Boc protected intermediate (270 mg, 86% yield). This material (260 mg, 0.660 mmol) was treated with a 1:1 mixture of CH_2Cl_2/TFA (1.32 mL) at 0 °C and stirred for 30 min at rt. Volatiles were evaporated and the product was obtained as a white solid (261 mg, 97%).

Mp = 128-129 °C; **δ_H /ppm** (500 MHz, d_4-MeOD): 8.27 (1H, app t, *J* = 1.8 Hz), 7.78 (1H, ddd, *J* = 8.1, 2.2, 1.1 Hz), 7.74 (1H, ddd, *J* =7.8, 1.6, 1.1 Hz), 7.42 (1H, ddd, *J* = 8.1, 7.8, 0.3 Hz), 3.90 (3H, s), 3.56 (2H, t, *J* = 6.5 Hz), 3.18 (2H, t, *J* = 6.5 Hz), 2.62 (2H, t, *J* = 6.5 Hz), 2.58 (2H, t, *J* = 6.5 Hz); **δ_C /ppm** (126 MHz, d_4-MeOD): 172.2 (Cx2), 168.2 (C), 140.2 (C), 132.0 (C), 130.1 (CH), 126.0 (CH), 125.5 (CH), 122.0 (CH), 52.7 (CH3), 37.2 (CH2), 37.1 (CH2), 36.8 (CH2), 32.8 (CH2); **ν_{max}/cm^{-1}:** 3308, 2928, 1702, 1657, 1283, 1118, 720; **HRMS** (ESI+) *m/z* found $[M+H]^+$ 294.1435, $C_{14}H_{20}N_3O_4^+$ required 294.1454.

Methyl 3-(3-(3-(((2-chloro-[1,1'-biphenyl]-4-yl)methyl)amino)propanamido)propanamido)benzoate 8

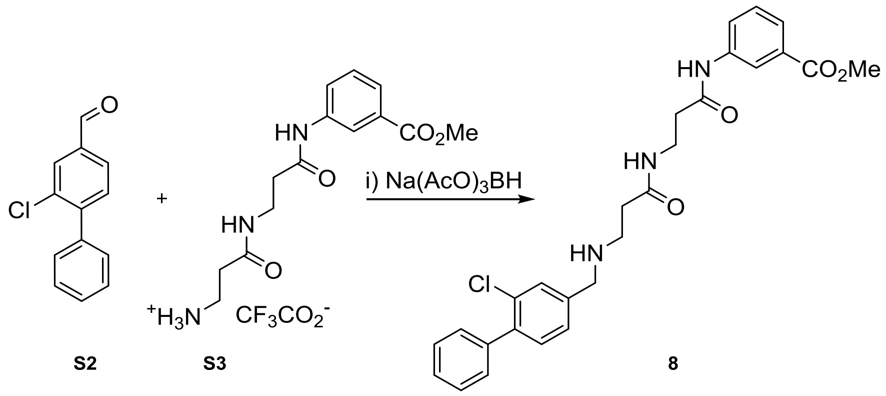

S2 **S3** **8**

A solution of ammonium salt **S3** (186 mg, 0.450 mmol) in methanol (1.25 mL) was treated with Et_3N (97.3 µL, 0.700 mmol) and aldehyde **S2** (75.8 mg, 0.350 mmol) and the mixture stirred at rt for 2 h. Sodium triacetoxyborohydride (104 mg, 0.490 mmol) was added in two portions with a 30 min interval and the reaction was stirred at rt for 18 h. The reaction mixture was poured into 2 M aqueous sodium carbonate and extracted with CH_2Cl_2 (3x). The combined organic extracts were dried ($MgSO_4$), concentrated under reduced pressure and purified by column chromatography eluting with a 98:2 to 94:6 CH_2Cl_2/MeOH gradient to give the free amine as a white solid (100 mg, 58%).

R$_f$ = 0.39 (CH_2Cl_2/MeOH 90:10); **δ_H /ppm** (400 MHz, *d*$_4$-MeOD): 8.26 (1H, t, *J* = 1.7 Hz), 7.82-7.77 (1H, m), 7.71 (1H, app d, *J* = 7.8 Hz), 7.47 (1H, d, *J* = 1.1 Hz), 7.43-7.32 (6H, m), 7.30 (1H, dd, *J* = 7.8, 1.3 Hz), 7.26 (1H, d, *J* = 7.8 Hz), 3.87 (3H, s), 3.77 (2H, s), 3.55 (2H, t, *J* = 6.4 Hz), 2.87 (2H, t, *J* = 6.7 Hz), 2.61 (2H, t, *J* = 6.4 Hz), 2.43 (2H, t, *J* = 6.6 Hz); **δ_C /ppm** (101 MHz, *d*$_4$-MeOD): 174.7 (C), 172.3 (C), 168.2 (C), 141.6 (C), 140.7 (C), 140.6 (C), 140.3 (C), 133.4 (C), 132.5 (CH), 131.9 (C), 130.8 (CH), 130.4 (CH), 130.0 (CH) 129.1 (CH), 128.6 (CH), 128.2 (CH), 125.9 (CH), 125.5 (CH), 121.9 (CH), 53.2 (CH2), 52.7 (CH3), 46.0 (CH2), 37.5 (CH2), 36.7 (CH2), 36.3 (CH2); **ν_{max}/cm^{-1}:** 2908, 2763, 1644, 1551, 1444, 1301, 1082, 763, 699; **HRMS** (ESI+) *m/z* found $[M+H]^+$ 494.1823, $C_{27}H_{29}N_3O_4{}^{35}Cl^+$ required 494.1847.

N-((2-chloro-[1,1'-biphenyl]-4-yl)methyl)-3-((3-((3-(methoxycarbonyl)phenyl)amino)-3-oxopropyl)amino)-3-oxopropan-1-aminium chloride (8_HCl)

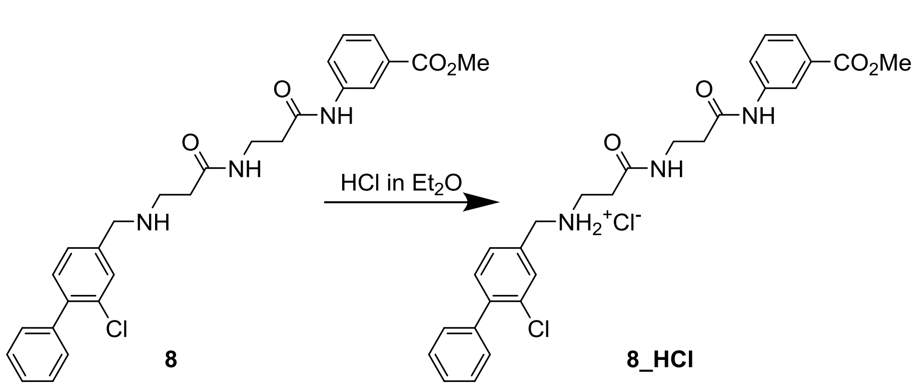

The amine **8** (55.3 mg, 0.112 mmol) was converted to the hydrochloride salt adding hydrochloric acid (0.560 ml, 2 M in diethyl ether) to a CH_2Cl_2 solution of the amine (0.4 M). The reaction was stirred for 1 h and the precipitate was filtered off and washed with diethyl ether to yield the product as a white solid (51.8 mg, 87%).

Mp = 229-232 °C; **δ_H /ppm** (500 MHz, *d*$_4$-MeOD): 8.28 (1H, t, *J* = 1.8 Hz), 7.77 (1H, ddd, *J* = 8.1, 2.3, 1.1 Hz), 7.73 (1H, ddd, *J* = 7.8, 1.6, 1.1 Hz), 7.69 (1H, app d, *J* = 1.6 Hz), 7.50 (1H, d, *J* = 7.8, 1.8 Hz), 7.47-7.43 (3H, m), 7.43-7.38 (4H, m), 4.28 (2H, s), 3.88 (3H, s), 3.58 (2H, t, *J* = 6.4 Hz), 3.34 (2H, t, *J* = 6.5 Hz), 2.69 (2H, t, *J* = 6.5 Hz), 2.64 (2H, t, *J* = 6.5 Hz); **δ_C /ppm** (126 MHz, *d*$_4$-MeOD): 172.3 (C), 172.1 (C), 168.2 (C), 143.2 (C), 140.2 (C), 139.8 (C), 134.1 (C), 133.3 (CH, C), 132.4 (CH), 132.0 (C), 130.3 (CH), 130.1 (CH), 129.7 (CH), 129.3 (CH), 129.1 (CH), 126.0 (CH), 125.5 (CH), 122.0 (CH), 52.7 (CH3), 51.2 (CH2), 44.7 (CH2), 37.2 (CH2), 36.9 (CH2), 31.7 (CH2); **ν_{max}/cm^{-1}:** 3293, 2929, 2779, 1728, 1664, 1640, 1542, 1427, 1297, 1234, 699.

3-(3-(3-(((2-Chloro-[1,1'-biphenyl]-4-yl)methyl)ammonio)propanamido)propanamido)benzoate (7)

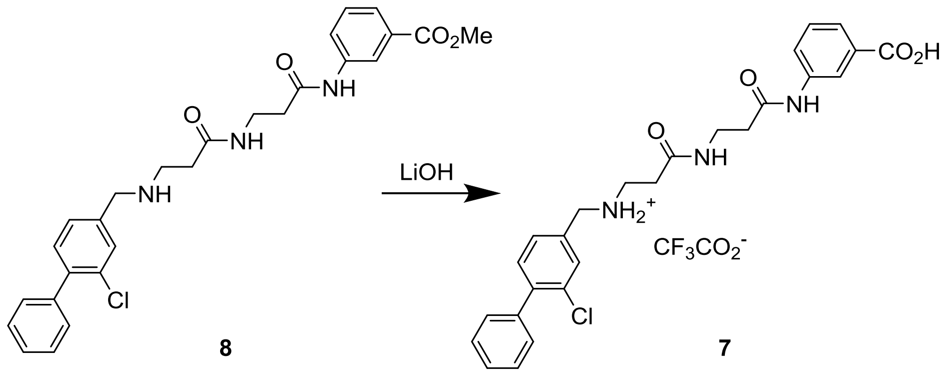

To a solution of the methyl ester **8** (59.3 mg, 0.120 mmol) in THF (2.40 mL) at 0 °C was added a 0.3 M aqueous LiOH solution (1.20 mL) and the mixture was stirred at rt until consumption of the starting material (~2 h). A 4N HCl solution in dioxane (3 equiv.) was added and after 10 min the solvent was removed under reduced pressure. The crude product was purified by HPLC t_r 9.12 min (method: 30-60% B, 20 min) (21.2 mg, 37%).

Mp = 96-98 °C; **δ_H /ppm** (500 MHz, d_4-MeOD): 8.25 (1H, t, *J* = 1.8 Hz), 7.78 (1H, ddd, *J* = 8.1, 2.1, 1.0 Hz), 7.75 (1H, app dt, *J* = 7.8, 1.3 Hz), 7.68 (1H, d, *J* = 1.6 Hz), 7.49 (1H, dd, *J* = 7.9, 1.8 Hz), 7.47-7.43 (3H, m), 7.43-7.38 (4H, m), 4.28 (2H, s), 3.58 (2H, t, *J* = 6.4 Hz), 3.32 (m, under the solvent peak), 2.67 (2H, t, *J* = 6.5 Hz), 2.63 (2H, t, *J* = 6.4 Hz); **δ_C /ppm** (126 MHz, d_4-MeOD): 172.3 (C), 172.1 (C), 169.4 (C), 143.3 (C), 140.1 (C), 139.8 (C), 134.2 (C), 133.3 (CH), 133.2 (C), 132.6 (C), 132.4 (CH), 130.3 (CH), 130.0 (CH), 129.6 (CH), 129.3 (CH), 129.1 (CH), 126.3 (CH), 125.4 (CH), 122.3 (CH), 51.2 (CH2), 44.7 (CH2), 37.2 (CH2), 36.9 (CH2), 31.7 (CH2); **ν_{max} /cm^{-1}:** 3081, 1657, 1553, 1442, 1186, 1134, 761; **HRMS** (ESI+) *m/z* found $[M+H]^+$ 480.1685, $C_{26}H_{27}N_3O_4{}^{35}Cl^+$ required 480.1669.

NMR spectra of compounds 7 and 8_HCl

^{1}H-NMR, d_4-MeOD, 500 MHz

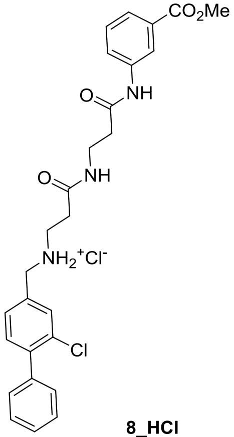

8_HCl

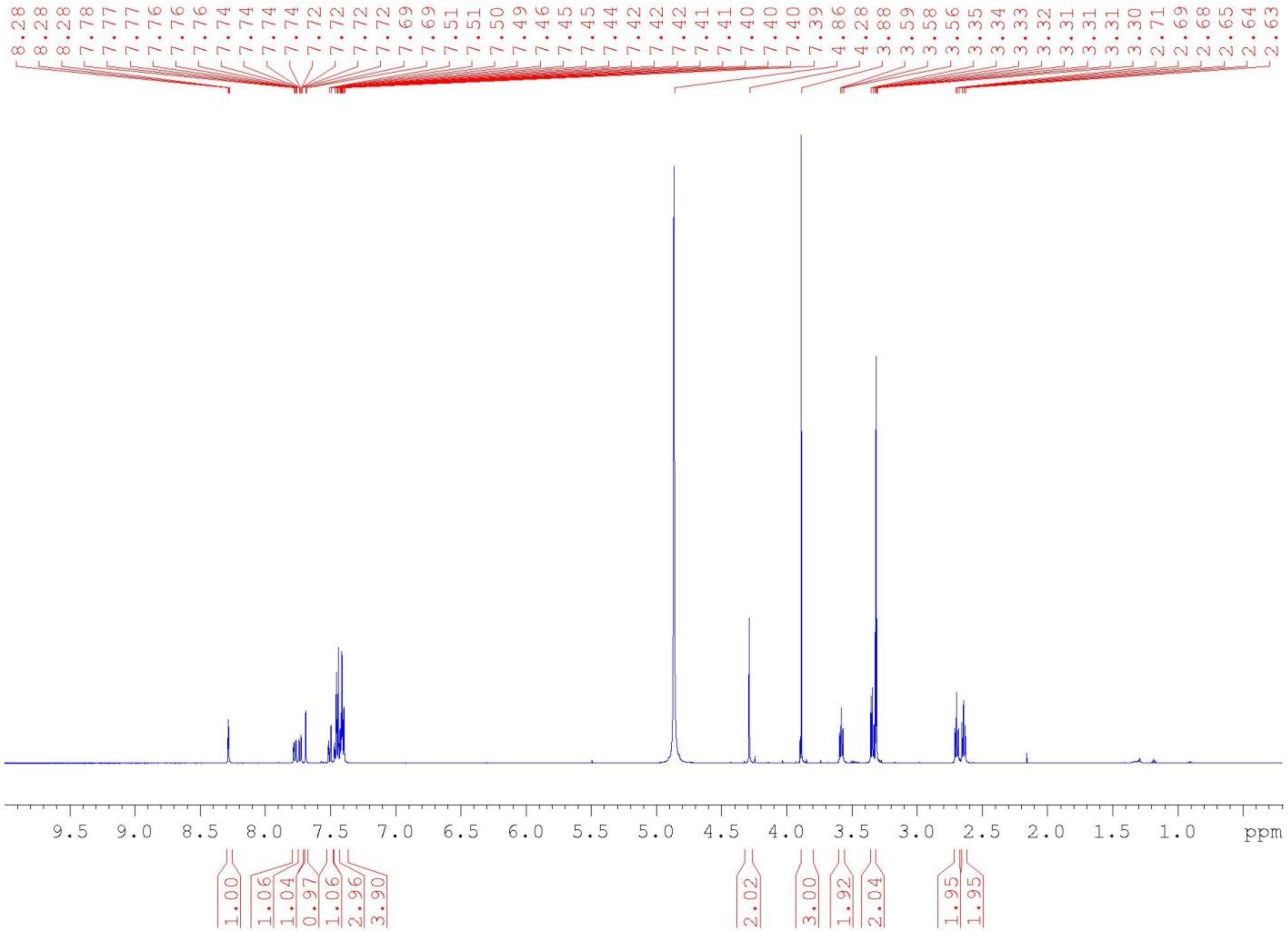

^{13}C-NMR, d_4-MeOD, 126 MHz

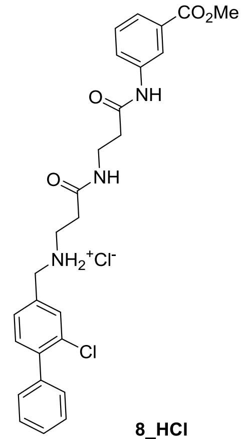

8_HCl

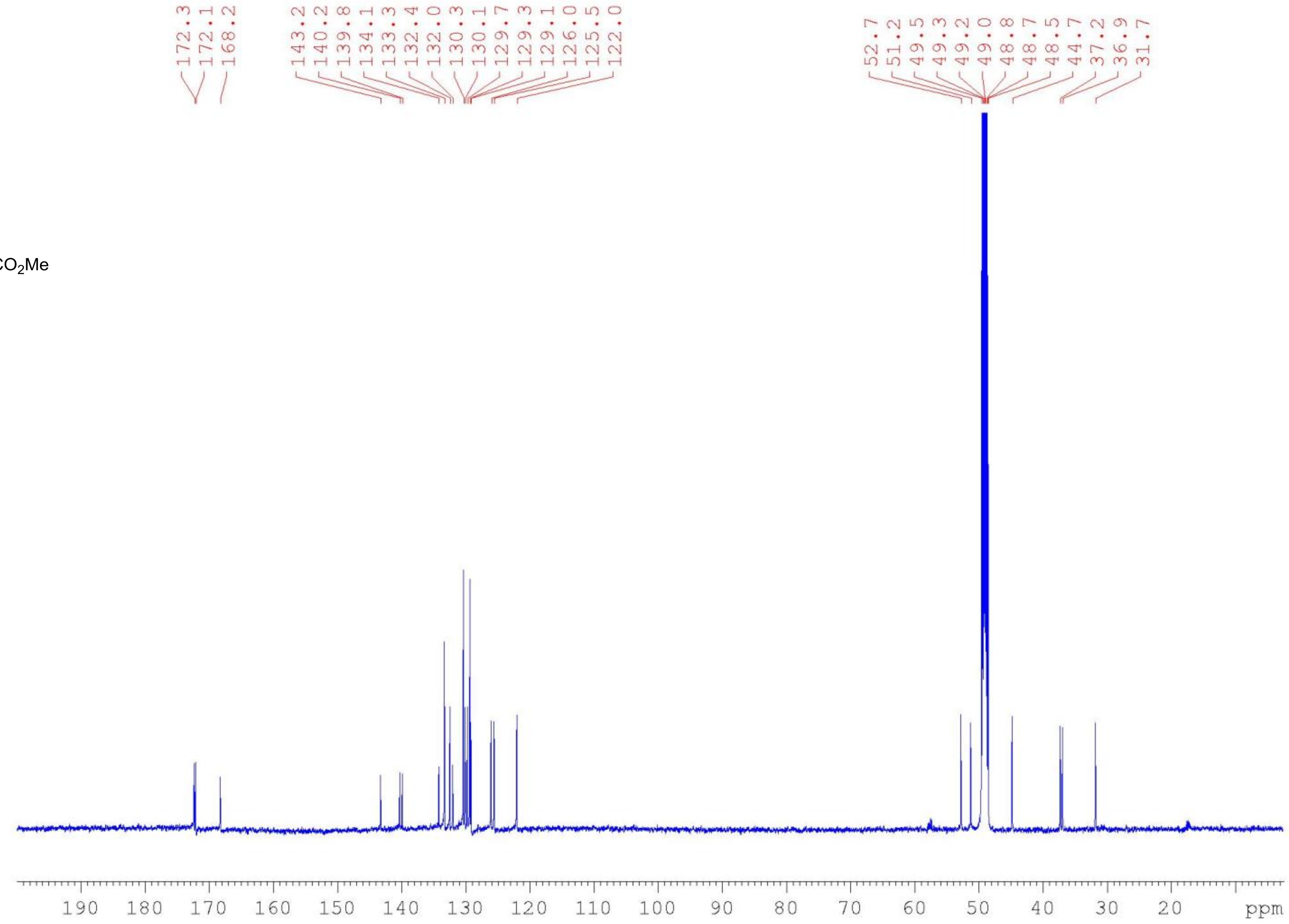

^{1}H-NMR, d_4-MeOD, 500 MHz

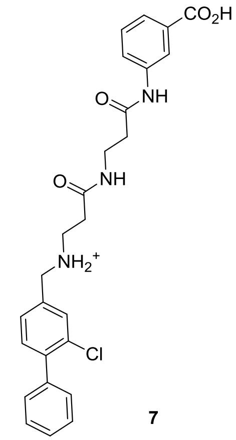

7

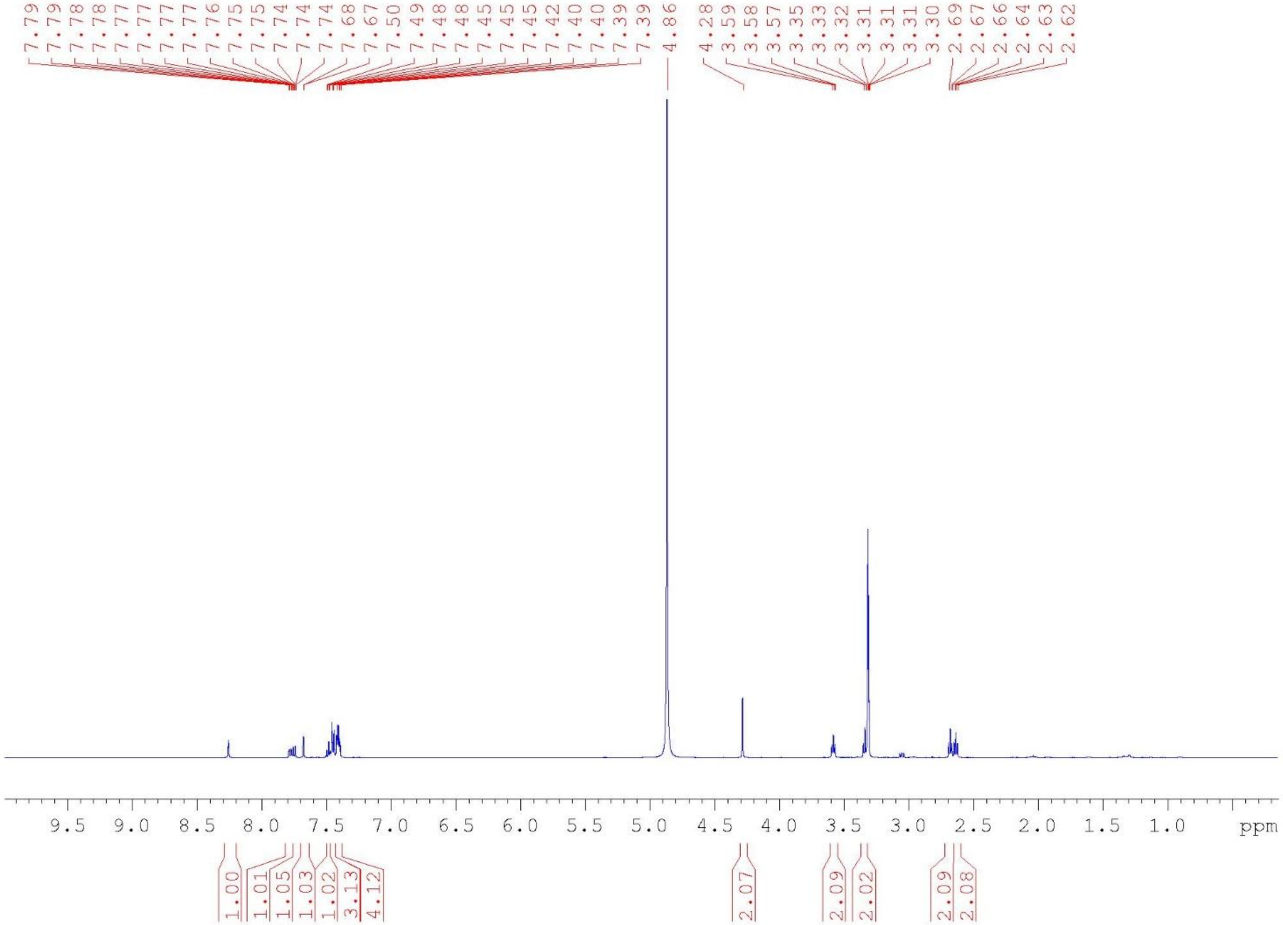

^{13}C-NMR, d_4-MeOD, 126 MHz

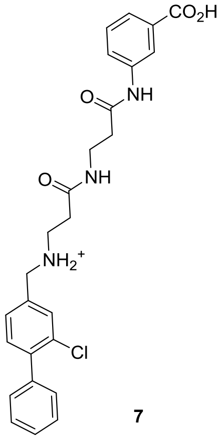

7

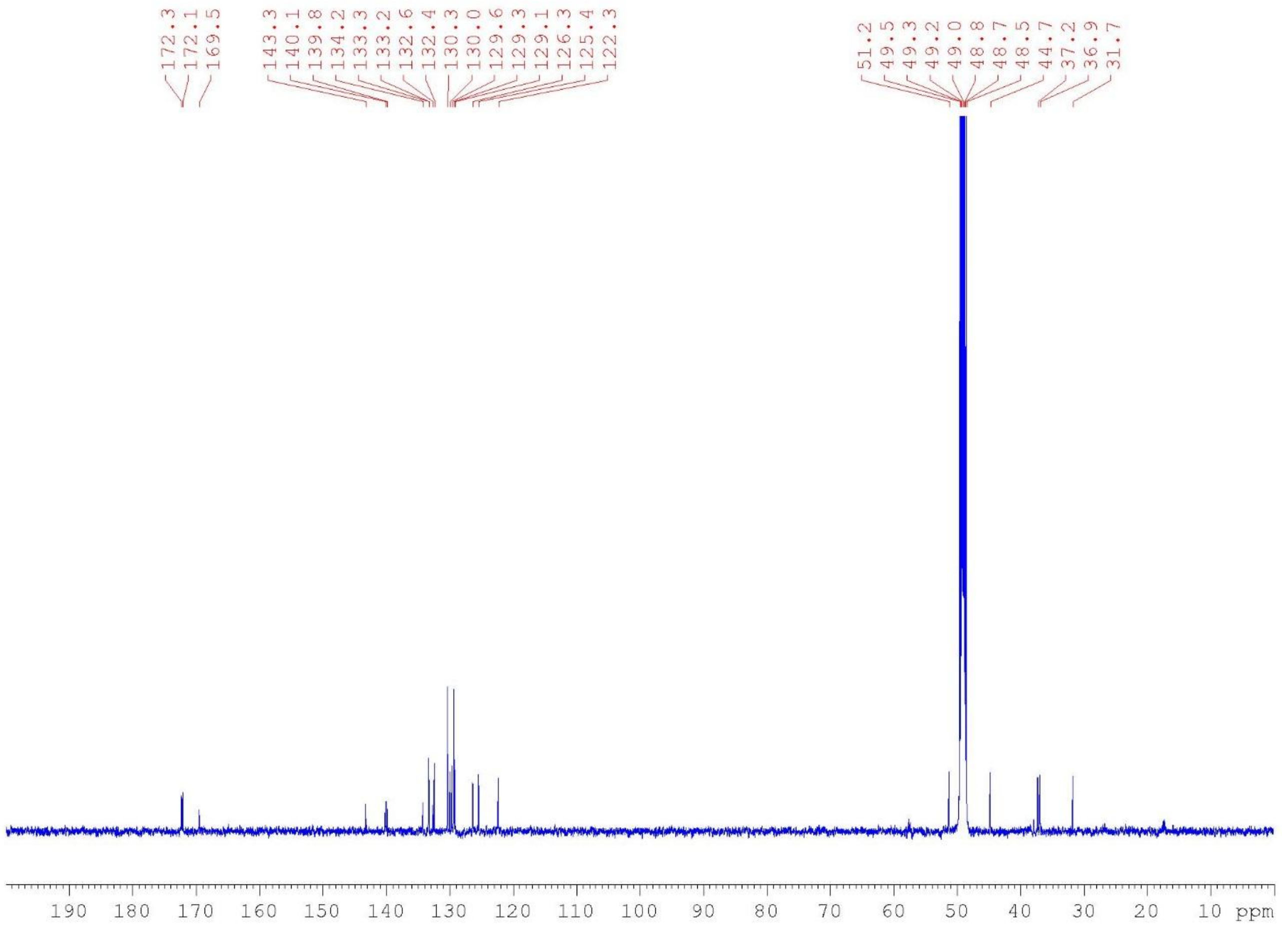

Supplement: Supplementary file 1 [file SC-007-C6SC02335E-s001.pdf]
